# Health Care Utilization in Adults With Congenital Heart Disease: Population‐Based Findings

**DOI:** 10.1002/bdr2.70063

**Published:** 2026-06-04

**Authors:** Tessa L. Crume, Amber D. Khanna, Alexandra Tillman, George K. Lui, Aida S. Soim, Daphne T. Hsu, Kristin Sommerhalter, Anaclare Sullivan, Jennifer S. Li, Alfred D′Ottavio, Wendy M. Book, Cheryl Raskind‐Hood, Kevin Whitehead, Nelangi Pinto, Marcia L. Feldkamp, Matthew R. Reeder, Sergey Krikov, Lorenzo D. Botto

**Affiliations:** ^1^ Colorado School of Public Health University of Colorado Anschutz Medical Center Aurora Colorado USA; ^2^ University of Colorado Anschutz Medical Campus Colorado School of Medicine, Departments of Internal Medicine and Pediatrics Aurora Colorado USA; ^3^ Stanford University School of Medicine Palo Alto California USA; ^4^ New York State Department of Health Albany New York USA; ^5^ Children's Hospital at Montefiore and Albert Einstein College of Medicine Bronx New York USA; ^6^ Duke University School of Medicine Durham North Carolina USA; ^7^ Duke Clinical Research Institute Durham North Carolina USA; ^8^ Emory University School of Medicine, and Rollins School of Public Health Atlanta Georgia USA; ^9^ University of Utah School of Medicine Salt Lake City Utah USA; ^10^ University of Washington Seattle WA USA

**Keywords:** access to care, adults, cardiology specialist, Charlson comorbidity index, CHD, healthcare utilization

## Abstract

**Background:**

Population‐based data on healthcare utilization in adults with congenital heart disease (CHD) are limited. We examined utilization patterns in a multi‐site, population‐based U.S. cohort of adults with CHD.

**Methods:**

This retrospective cohort linked health and administrative records from five regions (Colorado, North Carolina, Utah, metropolitan Atlanta, and New York). Adults aged 19–64 years with at least one CHD‐related ICD‐9‐CM code recorded during 2011–2013 were included. We evaluated inpatient, emergency department (ED), outpatient, and cardiology outpatient encounters, as well as cardiac diagnostic/imaging, therapeutic/interventional, and vascular procedures. Multivariable mixed‐effects logistic regression models compared utilization by CHD severity (severe vs. non‐severe), adjusting for sex, race/ethnicity, insurance, comorbidity, and site, and including age group as an effect modifier.

**Results:**

Over 3 years, this cohort of 18,877 adults (20.7% severe CHD), 45.9% had at least one hospitalization, 35.1% had an ED visit, 92.1% had an outpatient encounter, and 43.8% had a cardiology outpatient visit. Cardiac diagnostic/imaging procedures occurred in 67.8%, cardiac interventions in 20.3%, and vascular procedures in 10.5%. Severe CHD was associated with higher odds of cardiac procedures but lower odds of ED use than non‐severe CHD. Age modified these associations, and site‐level variation was marked, with cardiology visit rates ranging from 21% to 59%.

**Conclusions:**

Adults with CHD had high healthcare utilization, including frequent ED use, yet fewer than half saw a cardiology specialist, particularly at older ages. These findings highlight persistent gaps in lifelong care and substantial geographic variation, underscoring the need to improve access to specialized care for adults with CHD.

## 1 Introduction

Improved survival of infants with congenital heart disease (CHD) has led to a demographic shift in which adults with CHD outnumber children (Best and Rankin 2016; Marelli et al. 2007, 2014). An estimated 1.4 to 1.7 million adults live with CHD in the United States, a population that is expected to grow by 5% annually (Marelli et al. 2007, 2014). Despite major advances in diagnosis and treatment, CHD remains a lifelong condition with substantial comorbidity and complication risks and typically requires specialized care throughout adulthood (Raissadati et al. 2020; Müller et al. 2022; Dellborg et al. 2023). Yet current U.S. data on the lifetime healthcare burden in adults with CHD (ACHD) remain incomplete. Most studies have been limited to single centers or hospital systems or have focused on narrow utilization metrics (e.g., inpatient admissions only) (Agarwal, Sud, Khera, et al. 2016; Agarwal, Sud, and Menon 2016; Cedars et al. 2016; Gurvitz et al. 2007; Khan et al. 2021; Lu et al. 2014; Opotowsky et al. 2009; Steiner et al. 2018). Internationally, some population‐based analyses have quantified ACHD utilization (Mackie et al. 2007; Billett et al. 2008; Benderly et al. 2021; Dellborg et al. 2022), but differences in healthcare systems limit their applicability for U.S. healthcare planning, specialist workforce development, and quality improvement. To address this gap, the Centers for Disease Control and Prevention (CDC) established the multi‐state Lifespan Study (Glidewell et al. 2018) to conduct population‐based surveillance of health outcomes and resource use in individuals with CHD across five U.S. regions. A prior report from this study described healthcare use in adolescents with CHD (Lui et al. 2022). Here, we report on adults aged 19–64 years with CHD and their healthcare utilization across inpatient, emergency department, and outpatient settings, with a focus on age‐related patterns and engagement in specialty cardiology care.

## 2 Methods

### 2.1 Study Population

This analysis used data from the CDC‐funded CHD surveillance project, described in detail elsewhere (Glidewell et al. 2018). CHD cases were identified from five population‐based sites: statewide in Colorado, North Carolina, and Utah; five counties in metropolitan Atlanta (Georgia); and 11 counties in New York State.

Eligible individuals had at least one CHD‐related ICD‐9‐CM diagnosis code in the range 745.xx–747.xx recorded during January 1, 2011 through December 31, 2013. We applied standard exclusions for certain codes: congenital heart block (746.86), isolated patent foramen ovale versus secundum atrial septal defect (745.5) (Rodriguez et al. 2018), and other minor vascular anomalies unlikely to represent true CHD (e.g., isolated umbilical artery absence). Individuals had to be 19–64 years old at the first qualifying encounter (the first documented healthcare encounter associated with an eligible CHD code) and residents of the study area at that time.

### 2.2 CHD Severity Classification

CHD lesions were categorized based on ICD‐9‐CM codes as “severe CHD” versus “non‐severe CHD” using an established schema adapted from Marelli et al. (2014) Cases were classified as “severe CHD” if any diagnostic code indicated one of the following complex lesions: common truncus (745.0), transposition of the great vessels (745.1×), tetralogy of Fallot (745.2), common ventricle (745.3), endocardial cushion defects (745.6×, excluding 745.61), congenital atresia of pulmonary valve (746.01), congenital tricuspid atresia and stenosis (746.1), hypoplastic left heart syndrome (746.7), interruption of aortic arch (747.11), or total anomalous pulmonary venous connection (747.41). All other CHD diagnoses (primarily moderate or simple defects) were classified as “non‐severe CHD.”

### 2.3 Healthcare Utilization Patterns

Healthcare utilization was evaluated across four encounter types: inpatient, emergency department (ED), outpatient (all specialties), and cardiology outpatient encounters. Outpatient cardiology encounters were defined according to Lui GK et al. (generally, visits coded under cardiology or ACHD specialists) (Lui et al. 2022). Overlapping encounters were counted once and classified using a predefined hierarchy: (1) inpatient, (2) ED, (3) outpatient. For each encounter type, we measured the proportion of individuals with at least one encounter during 2011–2013 and the median (interquartile range) number of encounters among users. Cardiac‐related procedures were grouped into three categories using Agency for Healthcare Research and Quality Clinical Classification Software and CPT‐based groupings: (1) cardiac diagnostic/imaging, (2) cardiac therapeutic/interventional, and (3) vascular procedures (Lui et al. 2022). Full code lists are provided in Appendix 1. One study site (North Carolina) lacked the specialty identifiers needed to distinguish cardiology outpatient visits; therefore, analyses of cardiology‐specific outpatient utilization excluded that site (denominators adjusted accordingly in relevant statistics).

### 2.4 Insurance Type

Insurance at each encounter was categorized as private, Medicaid, Medicare, other government, self‐pay/uninsured, or other. For each individual, coverage over the study period was summarized into four mutually exclusive categories: private only, any public/other (≥ 1 Medicaid, Medicare, or other government encounter), self‐pay/uninsured/other (≥ 1 self‐pay or uninsured encounter and none with private/public), or unknown only (all entries “other” or missing).

### 2.5 Comorbidity Score

Comorbidity burden was measured using the Charlson Comorbidity Index (Charlson et al. 1987, 2022) adapted for ICD‐9/10 coding (Quan et al. 2005). We counted the number of distinct Charlson conditions (0 to ≥ 5) without weighting, as a measure of overall comorbidity.

### 2.6 Statistical Analysis

Sample size was determined by population‐based surveillance, including all eligible individuals in the catchment areas. We summarized cohort characteristics by site and CHD severity, using chi‐square tests for categorical variables and Kruskal–Wallis tests for continuous or count variables. For each utilization outcome, we calculated the proportion of patients with at least one encounter or procedure and the median (interquartile range) among users, overall and by CHD severity. Multivariable generalized linear mixed models with a logit link estimated adjusted odds ratios (ORs) and 95% confidence intervals for severe versus non‐severe CHD. Models adjusted for sex, race/ethnicity, insurance category, and Charlson comorbidity count and included a random intercept for site. Age group (19–24, 25–34, 35–44, 45–54, 55–64 years) was included as an interaction term with CHD severity; interaction was assessed using likelihood ratio tests. All analyses were performed using SAS 9.4 (SAS Institute, Cary, NC).

## 3 Results

### 3.1 Cohort Identification

**FIGURE 1:**
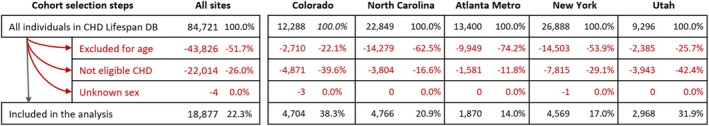
Selection of the analytic cohort of adults with congenital heart disease by study site, lifespan study. Note: The figure shows the number of identified cases at each site, exclusions by eligibility criteria, and the final number of adults with CHD included in the analytic cohort.

Across all sites, 84,721 individuals with a CHD‐related ICD‐9‐CM code were identified during 2011–2013 (Figure 1). After excluding individuals outside the 19–64 years of age range, those without an eligible CHD code, or with unknown sex, the final analytic cohort comprised 18,877 adults with CHD. Site‐specific cohort sizes are shown in Figure 1.

### 3.2 Cohort Characteristics

Age distribution varied by site (*p* < 0.0001). Overall, the largest age group was 25–34 years, with the proportion of young adults (19–24 years) ranging from 16.6% in Colorado to 21.7% in Atlanta, and the proportion of the oldest group (55–64 years) ranging from 15.3% in Atlanta to about 20% in other regions (Table 1). Severe CHD accounted for 20.7% of the cohort, with site‐specific proportions from 17.7% in Colorado to 29.2% in Atlanta (*p* = 0.07). Racial/ethnic composition also differed markedly (*p* < 0.0001): 60.5% of the cohort was non‐Hispanic White overall, ranging from 46.9% in New York to 75.5% in North Carolina; Black patients were most represented in Atlanta (31.7% vs. < 3% in Utah), and Hispanic patients in New York (28.8% vs. ~3% in North Carolina). Insurance coverage and comorbidity burden varied by region (both *p* < 0.0001): in New York and Utah, a majority had some form of public insurance (68.2% and 60.8%, respectively), and the proportion with at least one Charlson comorbidity (score ≥ 1) exceeded 50% in Atlanta (50.8%), New York (58.0%), and Utah (53.5%).

**TABLE 1:** Demographic and clinical characteristics of adults with congenital heart disease by study site, lifespan study.

	All sites *N* = 18,877		Colorado *N* = 4704		North Carolina *N* = 4766		Atlanta *N* = 1870^a^		New York *N* = 4569^b^		Utah *N* = 2968		χ^2^ *p*
	*N*	%	*N*	%	*N*	%	*N*	%	*N*	%	*N*	%	
*Sex*													0.001
Male	9075	48.1	2260	48.0	2516	52.8	810	43.3	2033	44.5	1456	49.1	
Female	9802	51.9	2444	52.0	2250	47.2	1060	56.7	2536	55.5	1512	50.9	
*Age group (years)*													< 0.0001
19–24	3655	19.4	780	16.6	923	19.4	405	21.7	935	20.5	612	20.6	
25–34	4500	23.8	1150	24.4	1057	22.2	502	26.8	1011	22.1	780	26.3	
35–44	3342	17.7	827	17.6	902	18.9	356	19	759	16.6	498	16.8	
45–54	3674	19.5	965	20.5	925	19.4	320	17.1	951	20.8	513	17.3	
55–64	3706	19.6	982	20.9	959	20.1	287	15.3	913	20	565	19	
*Race/ethnicity*													< 0.0001
Non‐Hispanic White	11,428	60.5	2531	53.8	3599	75.5	922	49.3	2142	46.9	2234	75.3	
Non‐Hispanic Black	2169	11.5	121	2.6	759	15.9	592	31.7	686	15	NR	NR	
Hispanic	2265	12.0	544	11.6	139	2.9	58	3.1	1315	28.8	209	7	
Asian/Pacific Islander	337	1.8	151	3.2	63	1.3	43	2.3	78	1.7	NR	NR	
Other Race	335	1.8	93	2	52	1.1	25	1.3	151	3.3	14	0.5	
Unknown	2343	12.4	1264	26.9	154	3.2	230	12.3	197	4.3	498	16.8	
*Insurance*													< 0.0001
Private only	7410	39.3	2749	58.4	2227	46.7	936	50.1	847	18.5	651	21.9	
Any public	8622	45.7	1750	37.2	1254	26.3	696	37.2	3117	68.2	1805	60.8	
Self‐pay/uninsured/other	1684	8.9	156	3.3	220	4.6	208	11.1	603	13.2	497	16.7	
Unknown	1161	6.2	49	1	1065	22.3	30	1.6	NR	NR	15	0.5	
*CHD severity*													0.0715
Severe	3911	20.7	834	17.7	1152	24.2	546	29.2	843	18.5	536	18.1	
Non‐severe	14,966	79.3	3870	82.3	3614	75.8	1324	70.8	3726	81.5	2432	81.9	
*Charlson comorbidity score*													< 0.0001
0	9346	49.5	2673	56.8	2454	51.5	920	49.2	1920	42	1379	46.5	
1	4595	24.3	1132	24.1	1151	24.2	405	21.7	1133	24.8	774	26.1	
2	2293	12.2	481	10.2	551	11.6	241	12.9	647	14.2	373	12.6	
3	1170	6.2	199	4.2	298	6.3	130	7.0	348	7.6	195	6.6	
4	669	3.5	116	2.5	150	3.1	74	4.0	222	4.9	107	3.6	
5+	804	4.3	103	2.2	162	3.4	100	5.3	299	6.5	140	4.7	

Abbreviations: CHD, congenital heart defect; NR, non‐reportable due to small number regulations.

^a^ The Atlanta (Georgia) study area includes the metropolitan counties of Clayton, Cobb, DeKalb, Fulton & Gwinnett.

^b^ The New York study area includes 11 counties: Allegany, Cattaraugus, Chautauqua, Erie, Genesee, Monroe, Niagara, Orleans, and Wyoming in the West; Bronx and Westchester in the Southeast.

Table 2 shows characteristics by CHD severity. Compared with adults with non‐severe CHD, those with severe CHD were younger (63.9% vs. 37.8% < 35 years; *p* < 0.0001), more often female (54.7% vs. 51.2%; *p* = 0.0001), more likely to have public insurance (48.9% vs. 44.8%; *p* = 0.005), and less likely to have comorbid conditions (55.8% vs. 47.9% with Charlson score 0; *p* < 0.0001).

**TABLE 2:** Baseline characteristics in adults (19–64 years) with congenital heart disease (CHD) by CHD severity, lifespan study.

	Severe *N* = 3911		Non‐severe *N* = 14,966		χ^2^ *p*
	*N*	%	*N*	%	
*Sex*					0.0001
Male	1773	45.3	7302	48.8	
Female	2138	54.7	7664	51.2	
*Age group*					< 0.0001
19–24	1112	28.4	2543	17.0	
25–34	1387	35.5	3113	20.8	
35–44	718	18.4	2624	17.5	
45–54	420	10.7	3254	21.7	
55–64	274	7.0	3432	22.9	
*Race/ethnicity*					< 0.0001
Non‐Hispanic White	2386	61.0	9042	60.4	
Non‐Hispanic Black	541	13.8	1628	10.9	
Hispanic	441	11.3	1824	12.2	
Asian/Pacific Islander	73	1.9	264	1.8	
Other	82	2.1	253	1.7	
Unknown	388	9.9	1955	13.1	
*Insurance*					0.0054
Private only	1425	36.4	5985	40.0	
Any public	1912	48.9	6710	44.8	
Self‐pay/uninsured/other	299	7.6	1385	9.3	
Unknown only	275	7.0	886	5.9	
*Charlson comorbidities*					< 0.0001
0	2181	55.8	7165	47.9	
1	855	21.9	3740	25.0	
2	451	11.5	1842	12.3	
3	207	5.3	963	6.4	
4	111	2.8	558	3.7	
5+	106	2.7	698	4.7	

### 3.3 Overall Healthcare Utilization

Over the three‐year period, 45.9% of adults with CHD had at least one hospitalization, with similar rates in severe and non‐severe CHD (45.8% vs. 46.0%; *p* = 0.87) (Table 3). Outpatient care was nearly universal: 92.1% had at least one outpatient encounter, with slightly higher rates in severe than non‐severe CHD (93.9% vs. 91.6%; *p* < 0.0001). Emergency department visits were common but less frequent in severe CHD (31.4% vs. 36.0%; *p* < 0.0001). Cardiology outpatient care was underutilized overall: 43.8% of adults (excluding the site without cardiology data) had at least one cardiology visit during 2011–2013. Cardiology visits were more frequent in severe than non‐severe CHD (51.1% vs. 42.0%; *p* < 0.0001), yet nearly half of adults in both groups did not see a cardiology specialist. Site‐level differences in cardiology follow‐up were large, ranging from about 21% of patients in New York to 59% in Colorado.

**TABLE 3:** Healthcare encounters and procedures in adults with congenital heart disease (CHD) by CHD severity, lifespan study.

	Total		Severe		Non‐severe	*p* ^a^	
	*N* = 18,877		*N* = 3911		*N* = 14,966		
*Inpatient encounters*							
With ≥ 1 encounter (*N*, %)	8672	45.9%	1792	45.8%	6880	46%	0.87
Frequency (median, IQR)^b^	2	1–4	2	1–4	2	1–3	0.24
*Outpatient encounters*							
With ≥ 1 encounter (*N*, %)	17,385	92.1%	3672	93.9%	13,713	91.6%	< 0.0001
Frequency^b^ (median, IQR)	7	3–20	7	3–18	8	3–21	0.79
*Outpatient cardiologist encounters* ^c^							
With ≥ 1 encounter (*N*, %)	6174	43.8%	1411	51.1%	4763	42.0%	< 0.0001
Frequency (median, IQR)^b^	2	1–4	2	1–4	2	1–4	< 0.0001
*Emergency department encounters*							
With ≥ 1 encounter (*N*, %)	6619	35.1%	1227	31.4%	5392	36.0%	< 0.0001
Frequency (Median, IQR)^b^	2	1–4	2	1–3	2	1–4	< 0.0001
*Cardiac procedures*							
With ≥ 1 diagnostic or imaging (*N*, %)	12,790	67.8%	2964	75.8%	9826	65.7%	< 0.0001
Frequency (median, IQR)^b^	2	1–4	3	1–5	2	1–4	< 0.0001
≥ 1 procedure or surgery (*N*, %)	3824	20.3%	888	22.7%	2936	19.6%	< 0.0001
Frequency (median, IQR)^b^	1	1–2	1	1–2	1	1–2	< 0.0001
≥ 1 vascular procedures (*N*, %)	1989	10.5%	430	11.0%	1559	10.4%	0.29
Frequency (median, IQR)^b^	1	1–1	1	1–1	1	1–1	0.29

*Note:* The table shows the number and proportion of individuals with at least one encounter or procedure during the three‐year study period. Among these individuals, the frequency of encounters or procedures is summarized by median and interquartile range (IQR).

^a^ compares severe versus non‐severe CHD (Chi square test for proportion, Kruskal–Wallis Test for encounter frequency).

^b^ frequency among individuals with at least one of the encounter/procedure types in the category examined.

^c^ One site excluded (North Carolina) for lack of information (denominators: total, *n* = 14,111; severe CHD, *n* = 2759; non‐severe CHD, *n* = 11,352).

*Cardiac Procedures* (Table 3). Utilization of cardiovascular procedures was also high and differed by CHD severity. Overall, 67.8% of adults underwent at least one cardiac diagnostic or imaging procedure during the study period, more often among those with severe than non‐severe CHD (75.8% vs. 65.7%; *p* < 0.0001). Cardiac therapeutic or interventional procedures occurred in 20.3% of adults (22.7% vs. 19.6% in severe vs. non‐severe CHD; *p* < 0.0001). Vascular procedures were less common (10.5% overall) and did not differ significantly by CHD severity (11.0% vs. 10.4%; *p* = 0.29).

### 3.4 Age‐Modifying Effects

**FIGURE 2:**
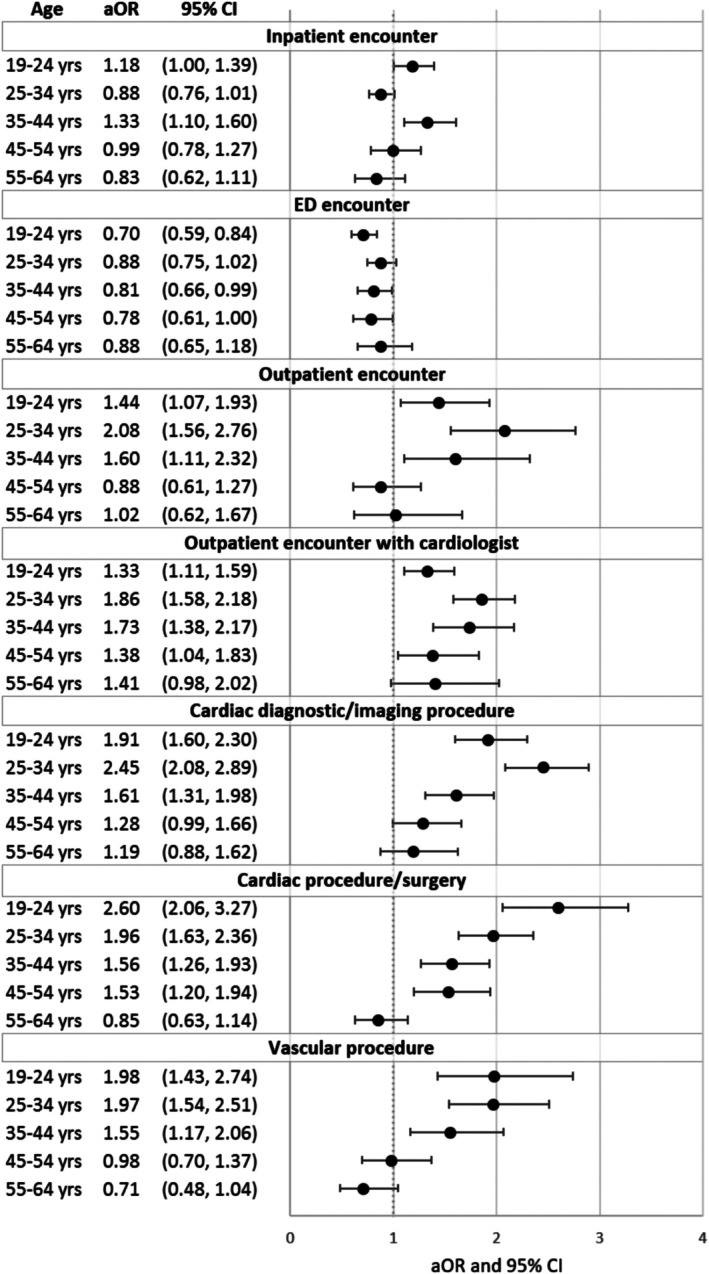
Adjusted odds of healthcare encounters and procedures in adults with severe versus non‐severe congenital heart disease by age group, lifespan study. Note: Odds ratios (ORs) with 95% confidence intervals are shown for each encounter and procedure type, comparing severe to non‐severe CHD across age groups. Models were adjusted for sex, race/ethnicity, insurance type, and Charlson comorbidity score, with study site included as a random intercept.

The association between CHD severity and utilization varied by age (Figure 2). In multivariable models, severe CHD was associated with higher odds of most encounter and procedure types among younger adults, with severity‐related differences decreasing with age. For example, among adults aged 19–24 years, severe CHD was associated with markedly higher odds of outpatient cardiology visits (OR > 2.0, 95% confidence interval excluding 1.0) and higher odds of any cardiac procedure compared with same‐age adults with non‐severe CHD. By ages 55–64 years, ORs for severe versus non‐severe CHD approached 1.0 for many outcomes, indicating attenuation of severity effects with age. In contrast, the inverse severity gradient in emergency department use (lower ED utilization in severe than non‐severe CHD) was evident across all age groups.

### 3.5 Site‐Specific Patterns

Healthcare utilization varied significantly by geographic site (Table S1). The proportion with at least one hospitalization ranged from about 27% in Colorado to 57% in North Carolina (overall 46%; *p* < 0.0001). Outpatient visit rates exceeded 90% in all regions, but median outpatient encounters per patient ranged from 4 in Atlanta to 14 in New York. Cardiology outpatient follow‐up showed the greatest variation, with site‐level cardiology visit rates ranging from 21% to 59% (*p* < 0.0001).

## 4 Discussion

This large population‐based study of U.S. adults with CHD demonstrates substantial lifelong healthcare use and important gaps in specialized care. Across five diverse regions, hospital‐based encounters were common, yet fewer than half of patients had any outpatient cardiology follow‐up. Over 3 years, nearly half were hospitalized at least once and about one‐third visited an emergency department, underscoring frequent acute‐care needs. Only 44% of patients (51% with severe CHD and 42% with non‐severe CHD) saw a cardiology specialist, well below guideline recommendations for lifelong follow‐up (Gurvitz et al. 2025; Stout et al. 2019), and indicates that most adults are not managed in specialized ACHD programs. Taken together with prior reports that 40%–50% of adults with CHD experience lapses in cardiology care longer than 3 years (Gurvitz et al. 2013), these findings highlight persistent challenges in maintaining lifelong specialized care (Khan et al. 2021).

An apparently paradoxical finding was that patients with non‐severe CHD had higher relative odds of emergency department use than those with severe CHD, suggesting an inverse severity gradient for emergency care. This pattern was most pronounced in mid‐adulthood (approximately ages 24–44) and persisted after adjustment for comorbidities and other covariates (Figure 2). Adults with milder CHD may be less engaged with specialized ACHD clinics and may rely more on episodic urgent care, whereas those with severe CHD often receive structured pediatric and ACHD follow‐up that may prevent some emergencies. Our results are consistent with the HEART‐ACHD study (Gurvitz et al. 2013), which found more frequent care lapses in patients with mild and moderate lesions than in those with complex CHD, and with studies linking rising ED use in ACHD populations to gaps in routine care and population growth (Agarwal et al. 2025). Evidence that routine ACHD outpatient care is associated with fewer emergent hospitalizations supports the interpretation that higher ED use among non‐severe CHD patients reflects inadequate outpatient coordination as well as disease burden and residual confounding.

Beyond emergency care, overall healthcare use was high across all settings. More than 90% of adults with CHD had at least one outpatient encounter over 3 years, two‐thirds underwent a cardiac diagnostic or imaging procedure, and about one in five had a major cardiac intervention or surgery. These findings underscore the resource‐intensive nature of CHD across the lifespan and show that care extends beyond cardiology clinics to general practitioners, emergency physicians, and other specialists. Our results are consistent with international data, including a population‐based study in Israel that reported 6–7‐fold higher hospitalization rates among adults with CHD compared with the general population and documented lower specialist follow‐up among women and minority groups (Benderly et al. 2021). We observed similar patterns, with women and patients in New York, which had a high Hispanic population, having particularly low cardiology follow‐up rates, suggesting opportunities to address population differences in access to ACHD care.

Age was an important determinant of utilization patterns. Older adults (55–64 years) were least likely to have cardiology visits, despite accumulating cardiovascular risk factors and CHD‐related sequelae. This paradox may reflect cohort effects. Older survivors grew up before dedicated ACHD services were established. Also, barriers such as retirement‐related insurance changes and distance from specialist centers may have played a role. These findings support extending ACHD care models beyond the pediatric‐to‐young‐adult transition into older adulthood and involving general cardiologists, internists, and geriatricians in co‐managing older patients with CHD.

Gaps in care during the transition from pediatric to adult services are also well documented. A meta‐analysis (Moons et al. 2021) estimated that about one quarter of adolescents and young adults with CHD experience discontinuity in cardiology care, with higher rates in the United States (34%) and among those with simple defects (34%) compared with moderate (26%) or complex CHD (22%). The same study noted that structured transition programs substantially reduced these lapses, yet reports a decade apart still show that nearly half of patients have gaps in care exceeding 3 years (Gurvitz et al. 2013; Zaidi et al. 2024). Given the elevated cardiovascular morbidity in young adults with CHD and the association between lapses in care and emergency presentations requiring urgent interventions (Saha et al. 2019; Oster et al. 2021; John et al. 2022), strengthening transition programs and sustained engagement strategies remain a critical priority.

We observed marked geographic differences in utilization and specialty engagement; for example, only about 21% of patients in New York saw a cardiology specialist compared with 59% in Colorado. These differences likely reflect variation in specialist availability and referral patterns. Many states are medically underserved for both primary and specialty care (U.S. Department of Health and Human Services, Health Resources and Services Administration, Bureau of Health Workforce 2025), and ACHD centers and board‐certified ACHD cardiologists remain relatively few and concentrated in major urban academic hubs (Ephrem 2025). Recent workforce analyses indicate that the current ACHD specialist supply can meet the needs of fewer than one‐third of adults with CHD, with projections suggesting that the shortfall will persist without substantial expansion of training and collaborative care models (Lin et al. 2025). Expanding outreach through satellite clinics, telemedicine, and regional centers of excellence, alongside investments in ACHD training and workforce growth, may help reduce geographic disparities in access to specialized care.

Our study has several limitations. First, reliance on clinical and administrative data introduces potential coding errors and omissions. Although we used a comprehensive case‐finding algorithm, excluded ambiguous codes such as ICD‐9745.5, and grouped lesions into broad severity categories, some misclassification of CHD diagnoses and severity is likely. We did not further subdivide the non‐severe group into simple and moderate lesions, which may have obscured additional heterogeneity but was consistent with prior methodology and allowed for stable estimates. Second, we lacked granular clinical information, such as functional status and reasons for emergency department visits, which limited our ability to distinguish encounters directly related to CHD from those attributable to other conditions. Third, site differences in data sources and healthcare systems may have influenced some results. One site did not contribute cardiology clinic data, and another contributed relatively fewer outpatient encounters, so cross‐site comparisons should be interpreted cautiously despite the use of site random effects. Fourth, our surveillance case definition required at least one encounter with a CHD code during 2011–2013. Individuals with CHD who did not seek care in that period, including some with very mild disease or those completely lost to follow‐up, were not captured, so utilization estimates apply to adults engaged with the healthcare system to some degree. Finally, the data reflect care from 2011–2013, before Medicaid expansion in many states, formal ACHD certification and center accreditation, the COVID‐19 pandemic, and widespread telehealth adoption; in the absence of more recent multi‐state surveillance, these data provide an important pre‐pandemic baseline but may not fully reflect current patterns.

Despite these limitations, the study has important strengths. It is one of the largest assembled cohorts of adults with CHD in the United States, includes multiple states with variable demographics, and uses a population‐based design that captures individuals across outpatient, emergency, and inpatient settings, including those outside major ACHD referral centers. We examined a broad range of utilization metrics and procedures within a single cohort and linked multiple data sources, providing a more comprehensive view of care than studies restricted to hospitalizations or insured populations. By adjusting for key covariates and accounting for clustering by site, our analysis addresses some underlying differences in case mix and regional practice.

In conclusion, in this multi‐state cohort of nearly 19,000 adults with CHD, we observed high inpatient and emergency care use, underuse of specialized cardiology outpatient care, and substantial geographic variation. These findings highlight critical gaps in the continuity of ACHD care and support development of targeted interventions, policies, and programs to improve lifelong follow‐up and access to adult congenital cardiology services—particularly for patients with moderate lesions and for older adults—with the goal of reducing preventable emergency visits and hospitalizations and improving quality of life in the growing population of adults living with CHD.

## Author Contributions

Dr. Tessa L. Crume, Dr. Amber K. Khanna, Alexandra Tillman, Dr. Matthew R. Reeder, Dr. Lorenzo Botto, Sergey Krikov, Dr. Kristin Sommerhalter, and Anaclare Sullivan had full access to all data in the study and take responsibility for the integrity of the data and the accuracy of the results. **Tessa L. Crume, Amber K. Khanna, Lorenzo D. Botto, Matthew Reeder, Sergey Krikov, Marcia L. Feldkamp:** analysis, interpretation of the data, and drafting of the manuscript. All authors: critical review of the manuscript for important intellectual content. **Alexandra Tillman, Sergey Krikov, Anaclare Sullivan, Kristin Sommerhalter: statistical analysis. Matthew R. Reeder, Jill Glidewell, Marcia L. Feldkamp, Lorenzo D. Botto:** administrative, technical, or material support. **Tessa L. Crume, Lorenzo D. Botto, Jill Glidewell:** supervision.

## Funding

This study was supported by the U.S. Centers for Disease Control and Prevention (Grant/Award Number CDC‐RFA‐DD15‐1506).

## Disclosure

The findings and conclusions in this report are those of the authors and do not necessarily represent the official position of the Centers for Disease Control and Prevention.

## Ethics Statement

The Institutional Review Boards from Emory University in Georgia (GA), the New York State Department of Health (NY), Duke University in North Carolina (NC), the University of Colorado (CO), and the University of Utah (UT) approved an analysis of de‐identified data. Each site's Institutional Review Board waived the requirement for informed consent due to the de‐identified data analysis.

## Supporting information


**Table S1:** Healthcare utilization by site.

**Appendix 1** Cardiac procedure categorization into cardiac diagnostic imaging (CDI), cardiac procedures/surgeries (CPS), and vascular procedures (VP) by International Classification of Diseases, Ninth Revision, Clinical Modification (ICD‐9‐CM) procedures codes and Current Procedural Terminology (CPT) codes and their descriptions.

## Data Availability

Research data are not shared.
