# Supplementary material for: Health Care Utilization in Adults With Congenital Heart Disease: Population‐Based Findings
Source: Birth Defects Res. 2026 Jun 4;118(6):e70063. doi: 10.1002/bdr2.70063 (PMC13238455; doi:10.1002/bdr2.70063)
Supplement: Supplementary file 1 — Table S1: Healthcare utilization by site. [file BDR2-118-e70063-s002.docx]

**Supplemental Table 1.** Healthcare utilization by site.

|  | All Sites  N = 18,877 | | Colorado  N = 4,704 | | N. Carolina  N = 4,766 | | Atlanta Metro*  N = 1,870 | | New York†  N = 4,569 | | Utah  N = 2,968 | | P-value‡ |
| --- | --- | --- | --- | --- | --- | --- | --- | --- | --- | --- | --- | --- | --- |
| Inpatient Encounters | | | | | | | | | | | | | |
| Cases with ≥ 1 (N, %) | 8,672 | 45.9% | 1,262 | 26.8% | 2,696 | 56.6% | 886 | 47.4% | 2,492 | 54.5% | 1,336 | 45.0% | <0.0001 |
| Frequency§ (Median, IQR) | 2 | 1 - 4 | 1 | 1 - 2 | 3 | 1 - 7 | 1 | 1 - 3 | 1 | 1 - 3 | 2 | 2 - 4 | <0.0001 |
| Outpatient Encounters | | | | | | | | | | | | | |
| Cases with ≥ 1 (N, %) | 17,385 | 92.1% | 4,315 | 91.7% | 4,496 | 94.3% | 1,482 | 79.3% | 4,185 | 91.6% | 2,907 | 97.9% | <0.0001 |
| Frequency§ (Median, IQR) | 7 | 3 - 20 | 5 | 2 - 11 | 6 | 3 - 14 | 4 | 2 - 19 | 14 | 4 - 32 | 13 | 5 - 27 | <0.0001 |
| Outpatient Cardiologist Encounters\|\| | | | | | | | | | | | | | |
| Cases with ≥ 1 (N, %) | 6,174 | 43.8% | 2,792 | 59.4% | NA | NA | 972 | 52.0% | 976 | 21.4% | 1,434 | 48.3% | <0.0001 |
| Frequency§ (Median, IQR) | 2 | 1 - 4 | 2 | 1 - 4 |  |  | 2 | 1 - 3 | 1 | 1 - 3 | 2 | 1 - 5 | <0.0001 |
| ED Encounters | | | | | | | | | | | | | |
| Cases with ≥ 1 (N, %) | 6,619 | 35.1% | 1,212 | 25.8% | 1,131 | 23.7% | 228 | 12.2% | 2,883 | 63.1% | 1,165 | 39.3% | <0.0001 |
| Frequency§ (Median, IQR) | 2 | 1 - 4 | 2 | 1 - 3 | 2 | 1 - 4 | 1 | 1 - 2 | 2 | 1 - 5 | 1 | 1 - 3 | <0.0001 |
| Cardiac Procedures | | | | | | | | | | | | | |
| Cases with ≥ 1 Diagnostic/Imaging Procedure (CDI) (N, %) | 12,790 | 67.8% | 2,591 | 55.1% | 2,636 | 55.3% | 1,559 | 83.4% | 3,661 | 80.1% | 2,343 | 78.9% | <0.0001 |
| CDI Frequency§ (Median, IQR) | 2 | 1 - 4 | 2 | 1 - 4 | 2 | 1 - 3 | 3 | 1 - 5 | 3 | 1 - 5 | 2 | 1 - 4 | <0.0001 |
| Cases with ≥ 1 Clinical Procedure/surgery (CPS) (N, %) | 3,824 | 20.3% | 639 | 13.6% | 1,172 | 24.6% | 367 | 19.6% | 1,077 | 23.6% | 569 | 19.2% | <0.0001 |
| CPS Frequency§ (Median, IQR) | 1 | 1 - 2 | 1 | 1 - 2 | 1 | 1 - 2 | 1 | 1 - 2 | 1 | 1 - 2 | 1 | 1 - 2 | <0.0001 |
| Cases with ≥ 1 Vascular Procedure (VP) (N, %) | 1,989 | 10.5% | 332 | 7.1% | 646 | 13.6% | 103 | 5.5% | 717 | 15.7% | 191 | 6.4% | <0.0001 |
| VP Frequency§ (Median, IQR) | 1 | 1 - 1 | 1 | 1 - 1 | 1 | 1 - 2 | 1 | 1 - 1 | 1 | 1 - 1 | 1 | 1 - 1 | 0.0017 |

Abbreviations: ED = emergency department; CDI = cardiac diagnostic/imaging procedures; CPS = cardiac procedure/surgery; VP = vascular procedure

* Atlanta Metro includes the metropolitan counties of Clayton, Cobb, DeKalb, Fulton & Gwinnett

† New York includes 11 counties: Allegany, Cattaraugus, Chautauqua, Erie, Genesee, Monroe, Niagara, Orleans, and Wyoming in the West; Bronx and Westchester in the Southeast

‡ Chi-square test for proportion comparison p-values; Kruskal-Wallis Test for encounter frequency distribution comparison p-value

§ Among those with at least one of the encounter type

|| Not including North Carolina, which did not have this data point
