# Supplementary material for: Health Care Utilization in Adults With Congenital Heart Disease: Population‐Based Findings
Source: Birth Defects Res. 2026 Jun 4;118(6):e70063. doi: 10.1002/bdr2.70063 (PMC13238455; doi:10.1002/bdr2.70063)
Supplement: Supplementary file 2 — Appendix 1 Cardiac procedure categorization into cardiac diagnostic imaging (CDI), cardiac procedures/surgeries (CPS), and vascular procedures (VP) by International Classification of Diseases, Ninth Revision, Clinical Modification (ICD‐9‐CM) procedures codes and Current Procedural Terminology (CPT) codes and their descriptions. [file BDR2-118-e70063-s001.pdf]

Appendix 1. Cardiac procedure categorization into cardiac diagnostic imaging (CDI), cardiac procedures/surgeries (CPS), and vascular procedures (VP) by International Classification of Diseases, Ninth Revision, Clinical Modification (ICD-9-CM) procedures codes and Current Procedural Terminology (CPT) codes and their descriptions.

	Procedure Code	Code Description
Cardiac Diagnostic Imaging (CDI)		
	International Classification of Diseases, Ninth Revision, Clinical Modification (ICD-9-CM)	
	88.72	Dx ultrasound-heart
	88.92	MRI chest & heart (begin 1986)
	89.41	Treadmill stress test
	89.42	Masters 2-step test
	89.43	Bicycle ergometer test
	89.44	Cardiac stress test nec
	89.50	Ambu cardiac monitoring (begin 1991)
	89.51	Rhythm electrocardiogram
	89.52	Electrocardiogram
	89.54	Electrocardiograph monit
	Current Procedural Terminology (CPT)	
	71275	Computed tomographic angiography, chest, without contrast material(s), followed by contrast material(s) and further sections, including image post#name?
	75552	Cardiac magnetic resonance imaging for morphology; without contrast material
	75553	Cardiac magnetic resonance imaging for morphology; with contrast material
	75554	Cardiac magnetic resonance imaging for function, with or without morphology; complete study
	75555	Cardiac magnetic resonance imaging for function, with or without morphology;limited study
	75556	Cardiac magnetic resonance imaging for velocity flow mapping
	76825	Echocardiography, fetal, cardiovascular system, real time with image documentation (2d) with or without m-mode recording;
	76826	Echocardiography, fetal, cardiovascular system, real time with image documentation (2d) with or without m-mode recording; follow-up or repeat study
	76827	Doppler echocardiography, fetal, cardiovascular system, pulsed wave and/or continuous wave with spectral display; complete
	76828	Doppler echocardiography, fetal, cardiovascular system, pulsed wave and/or continuous wave with spectral display; follow-up or repeat study
	76930	Ultrasonic guidance for pericardiocentesis, imaging supervision and interpretation
	76932	Ultrasonic guidance for endomyocardial biopsy, imaging supervision and interpretation
	78456	Acute venous thrombosis imaging, peptide
	78460	Myocardial perfusion imaging; (planar) single study, at rest or stress (exercise and/or pharmacologic), with or without quantification
	78464	Myocardial perfusion imaging; tomographic (spect), single study at rest or stress (exercise and/or pharmacologic), with or without quantification

	Procedure Code	Code Description
	78465	Myocardial perfusion imaging; tomographic (spect), mult. Studies at rest and/or stress (exercise and/or pharmacologic) and redistribution and/or rest injection, w/wo quant.
	78466	Myocardial imaging, infarct avid, planar; qualitative or quantitative
	78468	Myocardial imaging, infarct avid, planar; with ejection fraction by first pass technique
	78469	Myocardial imaging, infarct avid, planar; tomographic spect with or without quantification
	78472	Cardiac blood pool imaging, gated equilibrium; planar, single study at rest or stress (exercise and/or pharmacologic), wall motion study plus ejection fraction, wwo additional quantitative processing
	78473	Cardiac blood pool imaging, gated equilibrium; multiple studies, wall motion study plus ejection fraction, at rest and stress (exercise and/orpharmacologic), w w/o additional quantification
	78481	Cardiac blood pool imaging, (planar), first pass technique; single study, at rest or w/ stress (exercise and/or pharmacologic), wall motion studyplus ejection fraction, w/woquant.
	78483	Cardiac blood pool imaging, (planar), first pass tech.; mult. Studies, at rest & w/ stress (exercise and/or pharmacologic), wall motion study pluseject. Fraction, w/wo quantification
	78494	Cardiac blood pool imaging, gated equilibrium, spect, at rest, wall motion study plus ejection fraction, with or without quantitative processing
	78496	Cardiac blood pool imaging, gated equilibrium, single study, at rest, with right ventricular ejection fraction by first pass technique (list separately in addition to code for primary procedure)
	78499	Unlisted cardiovascular procedure, diagnostic nuclear medicine
	93000	Electrocardiogram, routine ecg with at least 12 leads; with interpretation and report
	93005	Electrocardiogram, routine ecg with at least 12 leads; tracing only, without interpretation and report
	93010	Electrocardiogram, routine ecg with at least 12 leads; interpretation and report only
	93012	Telephonic transmission of post-symptom electrocardiogram rhythm strip(s), 24-hour attended monitoring, per 30 day period of time;tracing only
	93014	Telephonic transmission of post-symptom electrocardiogram rhythm strip(s), 24-hour attended monitoring, per 30 day period of time;physician reviewwith interpretation and report only
	93015	Cardiovascular stress test using maximal or submaximal treadmill or bicycle exercise, continous electrocardiographic monitoring, pharmacologicstress; with phys supervision, interpret and report
	93016	Cardiovascular stress test using maximal or submaximal treadmill or bicycle exercise, continous electrocardiographic monitoring, pharmacologicstress; phys supervision only, w/o interpret & report
	93017	Cardiovascular stress test using maximal or submaximal treadmill or bicycle exercise, continous electrocardiographic monitoring, pharmacologicstress; tracing only, w/o interpret and report

	Procedure Code	Code Description
	93018	Cardiovascular stress test using maximal or submaximal treadmill or bicycle exercise, continous electrocardiographic monitoring, pharmacologicstress; interpretation and report only
	93025	Microvolt t-wave alternans for assessment of ventricular arrhythmias
	93040	Rhythm ecg, one to three leads; with interpretation and report
	93041	Rhythm ecg, one to three leads; tracing only without interpretation and report
	93042	Rhythm ecg, one to three leads; interpretation and report only
	93224	Electrocardiographic monitoring for 24 hours by continuous original ecg waveform record & storage w/ visual superimposition scan; inc recording, scan analysis w/ report, physician review & interpret
	93225	Electrocardiographic monitoring for 24 hours by continuous original ecg waveform record & storage w/ visual superimposition scan; recording (includes hook-up, recording, and disconnection)
	93226	Electrocardiographic monitoring for 24 hours by continuous original ecg waveform record & storage w/ visual superimposition scan; scanning analysiswith report
	93227	Electrocardiographic monitoring for 24 hours by continuous original ecg waveform record & storage w/ visual superimposition scan; physician reviewand interpretation
	93230	Electrocardiographic monitoring for 24 hours by continuous original ecg waveform record & storage w/o superimposition scan utilizing device capableof full mini printout; record & report
	93231	Electrocardiographic monitoring for 24 hours by continuous original ecg waveform record & storage w/o superimposition scan utilizing device capableof full mini printout; hook-up disconnect
	93232	Electrocardiographic monitoring for 24 hours by continuous original ecg waveform record & storage w/o superimposition scan utilizing device capableof full mini printout; microprocessor analysis
	93233	Electrocardiographic monitoring for 24 hours by continuous original ecg waveform record & storage w/o superimposition scan utilizing device capableof full mini printout; physician review & interpret
	93235	Electrocardiographic monitoring for 24 hours by continuous computer monitor & non-continuous record & real-time data analysis, full-sized tracings,w/ report, physician review & interpret
	93236	Electrocardiographic monitoring for 24 hours by continuous computer monitor & non-continuous record & real-time data analysis, full-sized tracings,possibly patient activated; real-time analysis
	93237	Electrocardiographic monitoring for 24 hours by continuous computer monitor & non-continuous record & real-time data analysis, full-sized tracings,possibly pt activated; physician rev & interpret
	93268	Patient demand single or multiple event recording with presymptom memory loop, 24-hour attended monitoring, per 30 day period of time; includestransmission, physician review and interpretation
	93270	Patient demand single or multiple event recording with presymptom memory loop, 24-hour attended monitoring, per 30 day period of time; recording (includes hook-up, recording, and disconnection)

	Procedure Code	Code Description
	93271	Patient demand single or multiple event recording with presymptom memory loop, 24-hour attended monitoring, per 30 day period of time; monitoring,receipt of transmissions, and analysis
	93272	Patient demand single or multiple event recording with presymptom memory loop, 24-hour attended monitoring, per 30 day period of time; physician review and interpretation only
	93278	Signal-averaged electrocardiography (saecg), with or without ecg
	93303	Transthoracic echocardiography for congential cardiac anomalies; complete
	93304	Transthoracic echocardiography for congential cardiac anomalies; follow-up or limited study
	93307	Echocardiography, transthoracic, real-time with image documentation (2d) with or without m-mode recording; complete
	93308	Echocardiography, real-time with image documentation (2d) with or without m-mode recording; follow-up or limited study
	93312	Echocardiography, transesophageal, real time with image documentation (2d) (with or without m-mode recording); including probe placement, imageacquisition, interpretation and report
	93313	Echocardiography, real time with image documentation (2d) (with or without m-mode recording), transesophageal; placement of transesophageal probeonly
	93314	Echocardiography, real time with image documentation (2d) (with or without m-mode recording), transesophageal; image acquisition, interpretation andreport only
	93315	Transesophageal echocardiography for congential cardiac anomalies; including probe placement, image acquisition, interpretation and report
	93316	Transesophageal echocardiography for congential cardiac anomalies; placement of transesophageal probe only
	93317	Transesophageal echocardiography for congential cardiac anomalies; image acquisition, interpretation and report only
	93318	Echocardiography,transesophageal monitor prposes,incl probe plcemnt,real time 2 dimenzional imge acquisit & interpret leading ongoing assess cardiac pump func & therapeutic msures immed time ba
	93320	Doppler echocardiography, pulsed wave and/or continuous wave with spectral display (list separately in addition to codes for echocardiographicimaging); complete
	93321	Doppler echocardiography, pulsed wave &/ continuous wave w spec disp (list sep in add to codes for echocardiographic imag); follow-up or lim study(list sep in add to codes for echocardiograph imag)
	93325	Doppler echocardiography color flow velocity mapping (list separately in addition to codes for echocardiography)
	93350	Echocardiography, transthoracic, real-time w image doc (2d, wwo m-mode rcrdng), during rest & cardiovasc stress tst using trdmill, bike and/orpharmacol induced stress, w interp & rpt
	93615	Esophageal recording of atrial electrogram with or without ventricular electrogram(s);
	93616	Esophageal recording of atrial electrogram with or without ventricular electrogram(s); with pacing

	Procedure Code	Code Description
	93660	Evaluation of cardiovascular function with tilt table evaluation, with continuous ecg monitoring and intermittent blood pressure monitoring, withor without pharmacological intervention
	93724	Electronic analysis of antitachycardia pacemaker (inc electrocardiographic recording, programming of device, induction & termination of tachycardiavia implanted pacemaker & interpret of recordings)
	Q0035	Cardiokymography
	S3902	Ballistocardiogram
	S3904	Masters two step
	S9025	Omnicardiogram/cardiointegram
Cardiac Surgery/Procedures (CPS)		
	ICD-9-CM	
	00.24	Ivus coronary vessels (begin 2004)
	00.50	Impla resynchr pacemaker w/0 defibri (begin 2002)
	00.51	Impla resynchronization defibrillator (begin 2002)
	00.52	Impl/repl transvenous lead lf ventri (begin 2002)
	00.53	Impl/repl pacemaker; plse genratr only (begin 2002)
	00.54	Impl/repl defibril generator only (begin 2002)
	00.56	Ins/rep impl sensor lead (begin 2006)
	00.57	Imp/rep subcue card dev (begin 2006)
	00.66	Ptca or coronary ather (begin 2005)
	17.51	Implant ccm
	17.52	Implant ccm pulse genrtr (begin 2009)
	17.55	Translum cor atherectomy (begin 2011)
	33.6	Comb heart/lung transpla (begin 1990)
	35.00	Closed valvotomy nos
	35.01	Closed aortic valvotomy
	35.02	Closed mitral valvotomy
	35.03	Closed pulmon valvotomy
	35.04	Closed tricusp valvotomy
	35.05	Endovas repl aortc valve (begin 2011)
	35.06	Trnsapcl rep aortc valve (begin 2011)
	35.07	Endovas repl pulm valve (begin 2011)
	35.08	Trnsapcl repl pulm valve (begin 2011)
	35.09	Endovas repl uns hrt vlv (begin 2011)
	35.10	Open valvuloplasty nos
	35.11	Opn aortic valvuloplasty
	35.12	Opn mitral valvuloplasty
	35.13	Opn pulmon valvuloplasty
	35.14	Opn tricus valvuloplasty

	Procedure Code	Code Description
	35.20	Replace heart valve nos
	35.21	Replace aort valv-tissue
	35.22	Replace aortic valve nec
	35.23	Replace mitr valv-tissue
	35.24	Replace mitral valve nec
	35.25	Replace pulm valv-tissue
	35.26	Replace pulmon valve nec
	35.27	Replace tric valv-tissue
	35.28	Replace tricusp valv nec
	35.31	Papillary muscle ops
	35.32	Chordae tendineae ops
	35.33	Annuloplasty
	35.34	Infundibulectomy
	35.35	Trabecul carneae cord op
	35.39	Tiss adj to valv ops nec
	35.41	Enlarge existing sep def
	35.42	Create septal defect
	35.50	Prosth rep hrt septa nos
	35.51	Pros rep atrial def-opn
	35.52	Pros repair atria def-cl
	35.53	Prost repair ventric def
	35.54	Pros rep endocar cushion
	35.55	Pros rep ventrc def-clos (begin 2006)
	35.60	Grft repair hrt sept nos
	35.61	Graft repair atrial def
	35.62	Graft repair ventric def
	35.63	Grft rep endocar cushion
	35.70	Heart septa repair nos
	35.71	Atria septa def rep nec
	35.72	Ventr septa def rep nec
	35.73	Endocar cushion rep nec
	35.81	Tot repair tetral fallot
	35.82	Total repair of tapvc
	35.83	Tot rep truncus arterios
	35.84	Tot cor transpos grt ves (begin 1988)
	35.91	Interat ven retrn transp
	35.92	Conduit rt vent-pul art
	35.93	Conduit left ventr-aorta
	35.94	Conduit artium-pulm art
	35.95	Heart repair revision

	Procedure Code	Code Description
	35.96	Perc heart valvuloplasty (begin 1986)
	35.97	Perc mtrl vlv repr w imp (begin 2010)
	35.98	Other heart septa ops
	35.99	Other heart valve ops
	36.01	Ptca-1 vessel w/o agent (begin 1986 end 2005)
	36.02	Ptca-1 vessel with agnt (begin 1986 end 2005)
	36.03	Open coronry angioplasty (begin 1986)
	36.04	Intrcoronry thromb infus (begin 1986)
	36.05	Ptca-multiple vessel (begin 1986 end 2005)
	36.06	Insert coron art stent (begin 1995)
	36.07	Insert drug-eluting crnry artry stnts (begin 2002)
	36.09	(oth)rem cor art obst(nec) (begin 1986)
	36.10	Aortocoronary bypass nos
	36.11	Aortocor bypas-1 cor art
	36.12	Aortocor bypas-2 cor art
	36.13	Aortocor bypas-3 cor art
	36.14	Aortcor bypas-4+ cor art
	36.15	1 int mam-cor art bypass
	36.16	2 int mam-cor art bypass
	36.17	Abd-coron art bypass (begin 1996)
	36.19	Hrt revas byps anas nec
	36.2	Arterial implant revasc
	36.3	Heart revascularizat nec (end 1998)
	36.31	Open chest transmyo revasc (begin 1998)
	36.32	Oth transmyo revasc (begin 1998)
	36.33	Endo transmyo revascular (begin 2006)
	36.34	Perc transmyo revascular (begin 2006)
	36.39	Other heart revasc (begin 1998)
	36.91	Coron vess aneurysm rep
	36.99	Heart vessel op nec
	37.0	Pericardiocentesis
	37.10	Incision of heart nos
	37.11	Cardiotomy
	37.12	Pericardiotomy
	37.20	Noninvas elect stimulatn (begin 2006)
	37.21	Rt heart cardiac cath
	37.22	Left heart cardiac cath
	37.23	Rt/left heart card cath
	37.24	Pericardial biopsy
	37.25	Cardiac biopsy

	Procedure Code	Code Description
	37.26	Cardiac electrophy stim (begin 1988)
	37.27	Cardiac mapping (begin 1988)
	37.28	Intracardiac echocardiography (begin 2001)
	37.29	Hrt/pericar dx proc nec
	37.31	Pericardiectomy
	37.32	Heart aneurysm excision
	37.33	Exc(/dest) oth hrt lesion (begin 1988)
	37.34	Cath ablation les heart (begin 1988)
	37.35	Partial ventriculectomy (begin 1997)
	37.36	Exc left atrial appendag (begin 2008)
	37.37	Exc/dest hrt les thrspc (begin 2010)
	37.4	Heart & pericard repair
	37.41	Impl cardiac support dev (begin 2005)
	37.49	Heart/pericard repr nec (begin 2005)
	37.5	Heart transplantation (end 2003)
	37.51	Heart transplantation (begin 2003)
	37.52	Implantation of total replacement heart syst (begin 2003)
	37.53	Replacement or repair of thoracic unit of to (begin 2003)
	37.54	Replacement or repair of other implantable c (begin 2003)
	37.55	Rem int bivent hrt sys (begin 2008)
	37.60	Imp bivn ext hrt ast sys (begin 2008)
	37.61	Pulsation balloon implan
	37.62	Implant hrt asst sys nec
	37.63	Replace hrt assist syst
	37.64	Remove heart assist sys
	37.65	Impl extern heart assist (begin 1995)
	37.66	Impl intern heart assist (begin 1995)
	37.67	Implant cardiomyostim sys (begin 1998)
	37.68	Percutan hrt assist syst (begin 2004)
	37.70	Int insert pacemak lead (begin 1987)
	37.71	Int insert lead in vent (begin 1987)
	37.72	Int inser lead atri-vent (begin 1987)
	37.73	Int inser lead in atrium (begin 1987)
	37.74	Int or repl lead epicar (begin 1987)
	37.75	Revision of lead (begin 1987)
	37.76	Repl tv atri-vent lead (begin 1987)
	37.77	Removal of lead w/o repl (begin 1987)
	37.78	Inser temp pacemaker sys (begin 1987)
	37.79	Revis or relocate pocket (begin 1987)
	37.80	Int or repl perm pacemkr (begin 1987)

	Procedure Code	Code Description
	37.81	Int insert 1-cham; non (begin 1987)
	37.82	Int insert 1-cham; rate (begin 1987)
	37.83	Int insert dual-cham dev (begin 1987)
	37.85	Repl pacem w 1-cham; non (begin 1987)
	37.86	Repl pacem 1-cham; rate (begin 1987)
	37.87	Repl pacem w dual-cham (begin 1987)
	37.89	Revise or remove pacemak (begin 1987)
	37.90	Ins left atr append dev (begin 2004)
	37.91	Opn chest cardiac massag
	37.92	Injection into heart
	37.93	Injection into pericard
	37.94	Implt/repl carddefib tot (begin 1986)
	37.95	Implt cardiodefib leads (begin 1986)
	37.96	Implt cardiodefib genatr (begin 1986)
	37.97	Repl cardiodefib leads (begin 1986)
	37.98	Repl cardiodefib genratr (begin 1986)
	37.99	Other heart/pericard ops
	38.04	Incision of aorta
	38.14	Endarterectomy of aorta
	38.15	Thoracic endarterectomy
	38.16	Abdominal endarterectomy
	38.26	Insrt prsr snsr w/o lead (begin 2011)
	38.34	Aorta resection & anast
	38.44	Resect abdm aorta w repl (begin 1986)
	38.64	Excision of aorta
	38.84	Occlude aorta nec
	39.21	Caval-pulmon art anastom
	39.23	Intrathoracic shunt nec
	39.61	Extracorporeal circulat
	39.62	Hypothermia w open heart
	39.63	Cardioplegia
	39.64	Intraop cardiac pacemak
	39.65	Extracorporeal memb oxy (begin 1988)
	39.66	Per cardiopulmon bypass (begin 1990)
	39.71	Endovascular implant in abdm aorta (begin 2000)
	39.73	Endo imp grft thor aorta (begin 2005)
	39.78	Endovas impln grft aorta (begin 2011)
	39.82	Imp/rep crtd sinus lead (begin 2010)
	39.83	Imp/rep crtd sinus gnrtr (begin 2010)
	39.84	Rev crtd sinus stm leads (begin 2010)

	Procedure Code	Code Description
	39.85	Rev crtd sinus pulse gen (begin 2010)
	39.86	Rem crtd sinus stm totl (begin 2010)
	39.87	Rem crtd sinus stm lead (begin 2010)
	39.88	Rem crtd sinus pulse gen (begin 2010)
	39.89	Oth cartd body/sinus op (begin 2010)
	39.96	Total body perfusion
	39.97	Other perfusion
	88.50	Angiocardiography nos
	88.52	Rt heart angiocardiogram
	88.53	Lt heart angiocardiogram
	88.54	Rt & lt heart angiocard
	88.55	Coronar arteriogr-1 cath
	88.56	Coronar arteriogr-2 cath
	88.57	Coronary arteriogram nec
	88.58	Negatve-contr cardiogram
	89.45	Pacemaker rate check
	89.46	Pacemaker wave form chck
	89.47	Pacemaker impedance chck
	89.48	Pacemaker volt threshold
	89.49	Pacemaker slew rate chck (begin 2004 end 1992)
	97.44	Nonop removal heart assist system (begin 2001)
	99.60	Cardiopulm resuscita nos
	99.61	Atrial cardioversion
	99.62	Heart countershock nec
	99.63	Closed chest card massag
	99.64	Carotid sinus stiumlat
	99.69	Cardiac rhythm conv nec
	CPT	
	00151	Transcatheter pulmonary valve implantation, percutaneous approach, including pre-stenting of the valve delivery site, when performed
	32160	Thoracotomy, major; with cardiac massage
	32658	Thoracoscopy, surgical; with removal of clot or foreign body from pericardial sac
	32659	Thoracoscopy, surgical; with creation of pericardial window or partial resection of pericardial sac for drainage
	32660	Thoracoscopy, surgical; with total pericardiectomy
	32661	Thoracoscopy, surgical; with excision of pericardial cyst, tumor, or mass
	33010	Pericardiocentesis; initial
	33011	Pericardiocentesis; subsequent
	33015	Tube pericardiostomy
	33020	Pericardiotomy for removal of clot or foreign body (primary procedure)

	Procedure Code	Code Description
	33025	Creation of pericardial window or partial resection for drainage
	33030	Pericardiectomy, subtotal or complete; without cardiopulmonary bypass
	33031	Pericardiectomy, subtotal or complete; with cardiopulmonary bypass
	33050	Excision of pericardial cyst or tumor
	33120	Excision of intracardiac tumor, resection with cardiopulmonary bypass
	33130	Resection of external cardiac tumor
	33200	Insertion of permanent pacemaker with epicardial electrode(s); by thoracotomy
	33201	Insertion of permanent pacemaker with epicardial electrode(s); by xiphoid approach
	33206	Insertion or replacement of permanent pacemaker with transvenous electrode(s); atrial
	33207	Insertion or replacement of permanent pacemaker with transvenous electrode(s); ventricular
	33208	Insertion or replacement of permanent pacemaker with transvenous electrode(s); atrial and ventricular
	33210	Insertion or replacement of temporary transvenous single chamber cardiac electrode or pacemaker catheter (separate procedure)
	33211	Insertion or replacement of temporary transvenous dual chamber pacing electrodes (separate procedure)
	33212	Insertion or replacement of pacemaker pulse generator only; single chamber, atrial or ventricular
	33213	Insertion or replacement of pacemaker pulse generator only; dual chamber
	33214	Upgrade of implanted pacemaker system, conversion single chamber sys to dual sys (inc removal previously placed pulse generator, test existinglead, insert new lead, insert new pulse generator)
	33215	Repositioning of previously implanted transvenous pacemaker or pacing cardioverter-defibrillator (right atrial or right ventricular) electrode
	33216	Insertion of a transvenous electrode; single chamber (one electrode) permanent pacemaker or single chamber pacing cardioverter-defibrillator
	33217	Insertion of a transvenous electrode; dual chamber (two electrodes) permanent pacemaker or dual chamber pacing cardioverter-defibrillator
	33218	Repair of single transvenous electrode for a single chamber, permanent pacemaker or single chamber pacing cardioverter-defibrillator
	33220	Repair of two transvenous electrodes for a dual chamber permanent pacemaker or dual chamber pacing cardioverter-defibrillator
	33222	Revision or relocation of skin pocket for pacemaker
	33223	Revision of skin pocket for single or dual chamber pacing cardioverter-defibrillator
	33224	Insrt, pacing electrde, card ven syst, left ventricular pacing, w attach previously placed pacemaker/pacing cardivrtr-defbr pulse gen (incl revis,pocket, rem, insrt &/ replcmnt, gen)
	33225	Insertion of pacing electrode, cardiac venous system, for left ventricular pacing, at time of insertion of pacing cardioverter-defibrillator orpacemaker pulse generator
	33226	Repositioning of previously implanted cardiac venous system (left ventricular) electrode (including removal, insertion and/or replacement of generator)

	Procedure Code	Code Description
	33233	Removal of permanent pacemaker pulse generator
	33234	Removal of transvenous pacemaker electrode(s); single lead system, atrial or ventricular
	33235	Removal of transvenous pacemaker electrode(s); dual lead system
	33236	Removal of permanent epicardial pacemaker and electrodes by thoracotomy; single lead system, atrial or ventricular
	33237	Removal of permanent epicardial pacemaker and electrodes by thoracotomy; dual lead system
	33238	Removal of permanent transvenous electrode(s) by thoracotomy
	33240	Insertion of single or dual chamber pacing cardioverter-defibrillator pulse generator
	33241	Subcutaneous removal of single or dual chamber pacing cardioverter-defibrillator pulse generator
	33243	Removal of single or dual chamber pacing cardioverter-defibrillator electrode(s); by thoracotomy
	33244	Removal of single or dual chamber pacing cardioverter-defibrillator electrode(s); by transvenous extraction
	33245	Insertion of epicardial single or dual chamber pacing cardioverter-defibrillator electrodes by thoracotomy
	33246	Insertion of epicardial single or dual chamber pacing cardioverter-defibrillator electrodes by thoracotomy; with insertion of pulse generator
	33249	Insertion or repositioning of electrode lead(s) for single or dual chamber pacing cardioverter-defibrillator and insertion of pulse generator
	33250	Operative ablation of supraventricular arrhythmogenic focus or pathway, tract(s) and/or focus; without cardiopulmonary bypass
	33251	Operative ablation of supraventricular arrhythmogenic focus or pathway, tract(s) and/or focus (foci); with cardiopulmonary bypass
	33253	Operative incisions and reconstruction of atria for treatment of atrial fibrillation or atrial flutter (eg, maze procedure)
	33261	Operative ablation of ventricular arrhythmogenic focus with cardiopulmonary bypass
	33282	Implantation of patient-activated cardiac event recorder
	33284	Removal of an implantable, patient-activated cardiac event recorder
	33300	Repair of cardiac wound; without bypass
	33305	Repair of cardiac wound; with cardiopulmonary bypass
	33310	Cardiotomy, exploratory (includes removal of foreign body); without bypass
	33315	Cardiotomy, exploratory (includes removal of foreign body); with cardiopulmonary bypass
	33330	Insertion of graft, aorta or great vessels; without shunt, or cardiopulmonary bypass
	33332	Insertion of graft, aorta or great vessels; with shunt bypass
	33335	Insertion of graft, aorta or great vessels; with cardiopulmonary bypass
	33361	Transcatheter aortic valve replacement femoral
	33362	Transcatheter aortic valve replacement femoral
	33363	Transcatheter aortic valve replacement femoral

	Procedure Code	Code Description
	33364	Transcatheter aortic valve replacement femoral
	33365	Transcatheter aortic valve replacement femoral
	33366	Transcatheter aortic valve replacement apical
	33400	Valvuloplasty, aortic valve; open, with cardiopulmonary bypass
	33401	Valvuloplasty, aortic valve; open, with inflow occlusion
	33403	Valvuloplasty, aortic valve; using transventricular dilation, with cardiopulmonary bypass
	33404	Construction of apical-aortic conduit
	33405	Replacement, aortic valve, with cardiopulmonary bypass; with prosthetic valve other than homograft or stentless valve
	33406	Replacement, aortic valve, with cardiopulmonary bypass; with allograft valve
	33410	Replacement, aortic valve, with cardiopulmonary bypass; with stentless tissue valve
	33411	Replacement, aortic valve; with aortic annulus enlargement, noncoronary cusp
	33412	Replacement, aortic valve; with transventricular aortic annulus enlargement (konno procedure)
	33413	Replacement, aortic valve; by translocation of autologous pulmonary valve with allograft replacement of pulmonary valve
	33414	Repair of left ventricular outflow tract obstruction by patch enlargement of the outflow tract
	33415	Resection or incision of subvalvular tissue for discrete subvalvular aortic stenosis
	33416	Ventriculomyotomy (-myectomy) for idiopathic hypertrophic subaortic stenosis (eg, asymmetric septal hypertrophy)
	33417	Aortoplasty (gusset) for supravalvular stenosis
	33420	Valvotomy, mitral valve; closed heart
	33422	Valvotomy, mitral valve; open heart, with cardiopulmonary bypass
	33425	Valvuloplasty, mitral valve, with cardiopulmonary bypass;
	33426	Valvuloplasty, mitral valve, with cardiopulmonary bypass; with prosthetic ring
	33427	Valvuloplasty, mitral valve, with cardiopulmonary bypass; radical reconstruction, with or without ring
	33430	Replacement, mitral valve, with cardiopulmonary bypass
	33460	Valvectomy, tricuspid valve, with cardiopulmonary bypass
	33463	Valvuloplasty, tricuspid valve; without ring insertion
	33464	Valvuloplasty, tricuspid valve; with ring insertion
	33465	Replacement, tricuspid valve, with cardiopulmonary bypass
	33468	Tricuspid valve repositioning and plication for ebstein anomaly
	33470	Valvotomy, pulmonary valve, closed heart; transventricular
	33471	Valvotomy, pulmonary valve, closed heart; via pulmonary artery
	33472	Valvotomy, pulmonary valve, open heart; with inflow occlusion
	33474	Valvotomy, pulmonary valve, open heart; with cardiopulmonary bypass
	33475	Replacement, pulmonary valve

	Procedure Code	Code Description
	33476	Right ventricular resection for infundibular stenosis, with or without commissurotomy
	33477	Transcatheter pulmonary valve implantation, percutaneous approach, including pre-stenting of the valve delivery site, when performed
	33478	Outflow tract augmentation (gusset), with or without commissurotomy or infundibular resection
	33496	Repair of non-structural prosthetic valve dysfunction with cardiopulmonary bypass (separate procedure)
	33500	Repair of coronary arteriovenous or arteriocardiac chamber fistula; with cardiopulmonary bypass
	33501	Repair of coronary arteriovenous or arteriocardiac chamber fistula; without cardiopulmonary bypass
	33502	Repair of anomalous coronary artery; by ligation
	33503	Repair of anomalous coronary artery; by graft, without cardiopulmonary bypass
	33504	Repair of anomalous coronary artery; by graft, with cardiopulmonary bypass
	33505	Repair of anomalous coronary artery; with construction of intrapulmonary artery tunnel (takeuchi procedure)
	33506	Repair of anomalous coronary artery; by translocation from pulmonary artery to aorta
	33508	Endoscopy, surgical, including video-assisted harvest of vein(s) for coronary artery bypass procedure (list separately in addition to code forprimary procedure)
	33542	Myocardial resection (eg, ventricular aneurysmectomy)
	33545	Repair of postinfarction ventricular septal defect, with or without myocardial resection
	33572	Coronary endarterectomy, open, any method, of left anterior descending, circumflex, or right coronary artery performed with coronary artery bypassgraft procedure, each vessel (list separately)
	33600	Closure of atrioventricular valve (mitral or tricuspid) by suture or patch
	33602	Closure of semilunar valve (aortic or pulmonary) by suture or patch
	33606	Anastomosis of pulmonary artery to aorta (damus-kaye-stansel procedure)
	33608	Repair of complex cardiac anomaly other than pulmonary atresia with ventricular septal defect by construction or replacement of conduit from rightor left ventricle to pulmonary artery
	33610	Repair of complex cardiac anomalies by surgical enlargement of ventricular septal defect
	33611	Repair of double outlet right ventricle with intraventricular tunnel repair;
	33612	Repair of double outlet right ventricle with intraventricular tunnel repair; with repair of right ventricular outflow tract obstruction
	33615	Repair of complex cardiac anomalies (eg, tricuspid atresia) by closure of atrial septal defect and anastomosis of atria or vena cava to pulmonaryartery (simple fontan procedure)
	33617	Repair of complex cardiac anomalies (eg, single ventricle) by modified fontan procedure

	Procedure Code	Code Description
	33619	Repair of single ventricle with aortic outflow obstruction and aortic arch hypoplasia (hypoplastic left heart syndrome) (eg, norwood procedure)
	33641	Repair atrial septal defect, secundum, with cardiopulmonary bypass, with or without patch
	33645	Direct or patch closure, sinus venosus, with or without anomalous pulmonary venous drainage
	33647	Repair of atrial septal defect and ventricular septal defect, with direct or patch closure
	33660	Repair of incomplete or partial atrioventricular canal (ostium primum atrial septal defect), with or without atrioventricular valve repair
	33665	Repair of intermediate or transitional atrioventricular canal, with or without atrioventricular valve repair
	33670	Repair of complete atrioventricular canal, with or without prosthetic valve
	33681	Closure of ventricular septal defect, with or without patch
	33684	Closure of ventricular septal defect, with or without patch with pulmonary valvotomy or infundibular resection (acyanotic)
	33688	Closure of ventricular septal defect, with or without patch with removal of pulmonary artery band, with or without gusset
	33690	Banding of pulmonary artery
	33692	Complete repair tetralogy of fallot without pulmonary atresia;
	33694	Complete repair tetralogy of fallot without pulmonary atresia; with transannular patch
	33697	Complete repair tetralogy of fallot with pulmonary atresia including construction of conduit from right ventricle to pulmonary artery and closureof ventricular septal defect;
	33702	Repair sinus of valsalva fistula, with cardiopulmonary bypass;
	33710	Repair sinus of valsalva fistula, with cardiopulmonary bypass; with repair of ventricular septal defect
	33720	Repair sinus of valsalva aneurysm, with cardiopulmonary bypass
	33722	Closure of aortico-left ventricular tunnel
	33730	Complete repair of anomalous venous return (supracardiac, intracardiac, or infracardiac types)
	33732	Repair of cor triatriatum or supravalvular mitral ring by resection of left atrial membrane
	33735	Atrial septectomy or septostomy; closed heart (blalock-hanlon type operation)
	33736	Atrial septectomy or septostomy; open heart with cardiopulmonary bypass
	33737	Atrial septectomy or septostomy; open heart, with inflow occlusion
	33750	Shunt; subclavian to pulmonary artery (blalock-taussig type operation)
	33755	Shunt; ascending aorta to pulmonary artery (waterston type operation)
	33762	Shunt; descending aorta to pulmonary artery (potts-smith type operation)
	33764	Shunt; central, with prosthetic graft
	33766	Shunt; superior vena cava to pulmonary artery for flow to one lung (classical glenn procedure)

	Procedure Code	Code Description
	33767	Shunt; superior vena cava to pulmonary artery for flow to both lungs (bidirectional glenn procedure)
	33770	Repair of transposition of the great arteries with ventricular septal defect and subpulmonary stenosis; without surgical enlargement of ventricular septal defect
	33771	Repair of transposition of the great arteries with ventricular septal defect and subpulmonary stenosis; with surgical enlargement of ventricular septal defect
	33774	Repair of transposition of the great arteries, atrial baffle procedure (eg, mustard or senning type) with cardiopulmonary bypass;
	33775	Repair of transposition of the great arteries, atrial baffle procedure (eg, mustard or senning type) with cardiopulmonary bypass; with removalofpulmonary band
	33776	Repair of transposition of the great arteries, atrial baffle procedure (eg, mustard or senning type) with cardiopulmonary bypass; with closureofventricular septal defect
	33777	Repair of transposition of the great arteries, atrial baffle procedure (eg, mustard or senning type) with cardiopulmonary bypass; with repair of subpulmonic obstruction
	33778	Repair of transposition of the great arteries, aortic pulmonary artery reconstruction (eg, jatene type)
	33779	Repair of transposition of the great arteries, aortic pulmonary artery reconstruction (eg, jatene type); with removal of pulmonary band
	33780	Repair of transposition of the great arteries, aortic pulmonary artery reconstruction (eg, jatene type); with closure of ventricular septal defect
	33781	Repair of transposition of the great arteries, aortic pulmonary artery reconstruction (eg, jatene type); with repair of subpulmonic obstruction
	33786	Total repair, truncus arteriosus (rastelli type operation)
	33788	Reimplantation of an anomalous pulmonary artery
	33800	Aortic suspension (aortopexy) for tracheal decompression (eg, for tracheomalacia) (separate procedure)
	33802	Division of aberrant vessel (vascular ring);
	33803	Division of aberrant vessel (vascular ring); with reanastomosis
	33813	Obliteration of aortopulmonary septal defect; without cardiopulmonary bypass
	33814	Obliteration of aortopulmonary septal defect; with cardiopulmonary bypass
	33820	Repair of patent ductus arteriosus; by ligation
	33822	Repair of patent ductus arteriosus; by division, under 18 years
	33824	Repair of patent ductus arteriosus; by division, 18 years and older
	33840	Excision of coarctation of aorta, with or without associated patent ductus arteriosus; with direct anastomosis
	33845	Excision of coarctation of aorta, with or without associated patent ductus arteriosus; with graft
	33851	Excision of coarctation of aorta, with or without associated patent ductus arteriosus; repair using either left subclavian artery or prostheticmaterial as gusset for enlargement
	33852	Repair of hypoplastic or interrupted aortic arch using autogenous or prosthetic material; without cardiopulmonary bypass
	33853	Repair of hypoplastic or interrupted aortic arch using autogenous or prosthetic material; with cardiopulmonary bypass

	Procedure Code	Code Description
	33860	Ascending aorta graft, with cardiopulmonary bypass, with or without valve suspension;
	33861	Ascending aorta graft, with cardiopulmonary bypass, with or without valve suspension; with coronary reconstruction
	33863	Ascending aorta graft, with cardiopulmonary bypass, with or without valve suspension; with aortic root replacement using composite prosthesis andcoronary reconstruction
	33870	Transverse arch graft, with cardiopulmonary bypass
	33875	Descending thoracic aorta graft, with or without bypass
	33877	Repair of thoracoabdominal aortic aneurysm with graft, with or without cardiopulmonary bypass
	33880	Endovascular repair procedures of the descending thoracic aorta
	33881	Endovascular repair procedures of the descending thoracic aorta
	33910	Pulmonary artery embolectomy; with cardiopulmonary bypass
	33915	Pulmonary artery embolectomy; without cardiopulmonary bypass
	33916	Pulmonary endarterectomy, with or without embolectomy, with cardiopulmonary bypass
	33917	Repair of pulmonary artery stenosis by reconstruction with patch or graft
	33918	Repair of pulmonary atresia with ventricular septal defect, by unifocalization of pulmonary arteries; without cardiopulmonary bypass
	33919	Repair of pulmonary atresia with ventricular septal defect, by unifocalization of pulmonary arteries; with cardiopulmonary bypass
	33920	Repair of pulmonary atresia with ventricular septal defect, by construction or replacement of conduit from right or left ventricle to pulmonaryartery
	33922	Transection of pulmonary artery with cardiopulmonary bypass
	33924	Ligation and takedown of a systemic-to-pulmonary artery shunt, performed in conjunction with a congenital heart procedure (list separately in addition to code for primary procedure)
	33925	Repair of pulmonary artery arborization anomalies by unifocalization; without cardiopulmonary bypass.
	33926	Repair of pulmonary artery arborization anomalies by unifocalization; with cardiopulmonary bypass.
	33930	Donor cardiectomy-pneumonectomy, with preparation and maintenance of allograft
	33935	Heart-lung transplant with recipient cardiectomy-pneumonectomy
	33940	Donor cardiectomy, with preparation and maintenance of allograft
	33945	Heart transplant, with or without recipient cardiectomy
	33960	Prolonged extracorporeal circulation for cardiopulmonary insufficiency; initial 24 hours
	33961	Prolonged extracorporeal circulation for cardiopulmonary insufficiency; each additional 24 hours (list separately in addition to code for primaryprocedure)
	33967	Insertion of intra-aortic balloon assist device, percutaneous
	33968	Removal of intra-aortic balloon assist device, percutaneous

	Procedure Code	Code Description
	33970	Insertion of intra-aortic balloon assist device through the femoral artery, open approach
	33971	Removal of intra-aortic balloon assist device including repair of femoral artery, with or without graft
	33973	Insertion of intra-aortic balloon assist device through the ascending aorta
	33974	Removal of intra-aortic balloon assist device from the ascending aorta, including repair of the ascending aorta, with or without graft
	33975	Insertion of ventricular assist device; extracorporeal, single ventricle
	33976	Insertion of ventricular assist device; extracorporeal, biventricular
	33977	Removal of ventricular assist device; extracorporeal, single ventricle
	33978	Removal of ventricular assist device; extracorporeal, biventricular
	33979	Insertion of ventricular assist device, implantable intracorporeal, single ventricle
	33980	Removal of ventricular assist device, implantable intracorporeal, single ventricle
	33999	Unlisted procedure, cardiac surgery
	34051	Embolectomy or thrombectomy, with or without catheter; innominate, subclavian artery, by thoracic incision
	34101	Embolectomy or thrombectomy, with or without catheter; axillary, brachial, innominate, subclavian artery, by arm incision
	34111	Embolectomy or thrombectomy, with or without catheter; radial or ulnar artery, by arm incision
	34151	Embolectomy or thrombectomy, with or without catheter; renal, celiac, mesentery, aortoiliac artery, by abdominal incision
	34401	Thrombectomy, direct or with catheter; vena cava, iliac vein, by abdominal incision
	34490	Thrombectomy, direct or with catheter; axillary and subclavian vein, by arm incision
	34501	Valvuloplasty, femoral vein
	34502	Reconstruction of vena cava, any method
	34510	Venous valve transposition, any vein donor
	34530	Saphenopopliteal vein anastomosis
	34800	Endovascular repair of infrarenal abdominal aortic aneurysm or dissection; using aorto-aortic tube prosthesis
	34802	Endovascular repair of infrarenal abdominal aortic aneurysm or dissection; using modular bifurcated prosthesis (one docking limb)
	34804	Endovascular repair of infrarenal abdominal aortic aneurysm or dissection; using unibody bifurcated prosthesis
	34808	Endovascular placement of iliac artery occlusion device (list separately in addition to code for primary procedure)
	34812	Open femoral artery exposure for delivery of endovascular prosthesis, by groin incision, unilateral
	34813	Placement of femoral-femoral prosthetic graft during endovascular aortic aneurysm repair (list separately in addition to code for primary procedure)
	34820	Open iliac artery exposure for delivery of endovascular prosthesis or iliac occlusion during endovascular therapy, by abdominal or retroperitonealincision, unilateral

	Procedure Code	Code Description
	34825	Placement of proximal or distal extension prosthesis for endovascular repair of infrarenal abdominal aortic or iliac aneurysm, false aneurysm,ordissection; initial vessel
	34826	Placement, proximal/distal exten prosthesis, endovascular repair, infrarenal abdominal aortic/iliac aneurysm, false aneurysm,/dissection; ea addition vessel (list separately addition cd, 1 procedu
	34830	Open repair of infrarenal aortic aneurysm or dissection, plus repair of associated arterial trauma, following unsuccessful endovascular repair;tube prosthesis
	34831	Open repair of infrarenal aortic aneurysm or dissection, plus repair of associated arterial trauma, following unsuccessful endovascular repair;aorto-bi-iliac prosthesis
	34832	Open repair of infrarenal aortic aneurysm or dissection, plus repair of associated arterial trauma, following unsuccessful endovascular repair;aorto-bifemoral prosthesis
	34833	Open iliac artery exposure with creation, conduit for delivery, infrarenal aortic or iliac endovascular prosthesis, abdominal or retroperitoneal incision, unilateral
	34834	Open brachial artery exposure to assist in the deployment of infrarenal aortic or iliac endovascular prosthesis by arm incision, unilateral
	34900	Endovascular graft replacement for repair of iliac artery (eg, aneurysm,pseudoaneurysm, arteriovenous malformation, trauma)
	35011	Direct repair of aneurysm, pseudoaneurysm, or excision and graft insertion, with or without patch graft; for aneurysm and associated occlusivedisease, axillary-brachial artery, by arm incision
	35013	Direct repair of aneurysm, pseudoaneurysm, or excision and graft insertion, with or without patch graft; for ruptured aneurysm, axillary-brachialartery, by arm incision
	35021	Direct repair,aneurysm,pseudoaneurysm, or excision&graft insertion, with/without patch graft;aneurysm, pseudoaneurysm&associated occlusive disease, innominate, subclavian artery, thoracic incision
	35022	Direct repair of aneurysm, pseudoaneurysm, or excision and graft insertion, with or without patch graft; for ruptured aneurysm, innominate, subclavian artery, by thoracic incision
	35045	Direct repair of aneurysm, pseudoaneurysm, or excision and graft insertion, with or without patch graft; for aneurysm, pseudoaneurysm, and associated occlusive disease, radial or ulnar artery
	35081	Direct repair of aneurysm, pseudoaneurysm, or excision and graft insertion, with or without patch graft; for aneurysm, pseudoaneurysm, and associated occlusive disease, abdominal aorta
	35082	Direct repair of aneurysm, pseudoaneurysm, or excision and graft insertion, with or without patch graft; for ruptured aneurysm, abdominal aorta
	35091	Direct repair, aneurysm, pseudoaneurysm/excision&graft insertion, with/without patch graft; for aneurysm, pseudoaneurysm,&associated occlusivedisease, abdominal aorta involving visceral vessels
	35092	Direct repair of aneurysm, pseudoaneurysm, or excision and graft insertion, with or without patch graft; for ruptured aneurysm, abdominal aortainvolving visceral vessels

	Procedure Code	Code Description
	35102	Direct repair, aneurysm, pseudoaneurysm, or excision&graft insertion, with or without patch graft; for aneurysm, pseudoaneurysm,&associated occlusive disease, abdominal aorta involving iliac vessels
	35103	Direct repair of aneurysm, pseudoaneurysm, or excision and graft insertion, with or without patch graft; for ruptured aneurysm, abdominal aortainvolving iliac vessels
	35111	Direct repair of aneurysm, pseudoaneurysm, or excision and graft insertion, with or without patch graft; for aneurysm, pseudoaneurysm, and associated occlusive disease, splenic artery
	35112	Direct repair of aneurysm, pseudoaneurysm, or excision and graft insertion, with or without patch graft; for ruptured aneurysm, splenic artery
	35121	Direct repair, aneurysm, pseudoaneurysm/excision&graft insertion, with/without patch graft; for aneurysm, pseudoaneurysm&associated occlusive disease, hepatic, celiac, renal/mesenteric artery
	35122	Direct repair of aneurysm, pseudoaneurysm, or excision and graft insertion, with or without patch graft; for ruptured aneurysm, hepatic, celiac, renal, or mesenteric artery
	35131	Direct repair of aneurysm, pseudoaneurysm, or excision and graft insertion, with or without patch graft; for aneurysm, pseudoaneurysm, and associated occlusive disease, iliac artery
	35132	Direct repair of aneurysm, pseudoaneurysm, or excision and graft insertion, with or without patch graft; for ruptured aneurysm, iliac artery
	35141	Direct repair of aneurysm, pseudoaneurysm, or excision and graft insertion, with or without patch graft; for aneurysm, pseudoaneurysm, and associated occlusive disease, common femoral artery
	35142	Direct repair of aneurysm, pseudoaneurysm, or excision and graft insertion, with or without patch graft; for ruptured aneurysm, common femoralartery
	35151	Direct repair of aneurysm, pseudoaneurysm, or excision and graft insertion, with or without patch graft; for aneurysm, pseudoaneurysm, and associated occlusive disease, popliteal artery
	35152	Direct repair of aneurysm, pseudoaneurysm, or excision and graft insertion, with or without patch graft; for ruptured aneurysm, popliteal artery
	35161	Direct repair of aneurysm, pseudoaneurysm, or excision and graft insertion, with or without patch graft; for aneurysm, pseudoaneurysm, and associated occlusive disease, other arteries
	35162	Direct repair of aneurysm, pseudoaneurysm, or excision and graft insertion, with or without patch graft; for ruptured aneurysm, other arteries
	35182	Repair, congenital arteriovenous fistula; thorax and abdomen
	35184	Repair, congenital arteriovenous fistula; extremities
	35189	Repair, acquired or traumatic arteriovenous fistula; thorax and abdomen
	35190	Repair, acquired or traumatic arteriovenous fistula; extremities
	35201	Repair blood vessel, direct; neck
	35206	Repair blood vessel, direct; upper extremity
	35207	Repair blood vessel, direct; hand, finger
	35211	Repair blood vessel, direct; intrathoracic, with bypass

	Procedure Code	Code Description
	35216	Repair blood vessel, direct; intrathoracic, without bypass
	35221	Repair blood vessel, direct; intra-abdominal
	35226	Repair blood vessel, direct; lower extremity
	35236	Repair blood vessel with vein graft; upper extremity
	35241	Repair blood vessel with vein graft; intrathoracic, with bypass
	35246	Repair blood vessel with vein graft; intrathoracic, without bypass
	35251	Repair blood vessel with vein graft; intra-abdominal
	35256	Repair blood vessel with vein graft; lower extremity
	35266	Repair blood vessel with graft other than vein; upper extremity
	35271	Repair blood vessel with graft other than vein; intrathoracic, with bypass
	35276	Repair blood vessel with graft other than vein; intrathoracic, without bypass
	35281	Repair blood vessel with graft other than vein; intra-abdominal
	35286	Repair blood vessel with graft other than vein; lower extremity
	35311	Thromboendarterectomy, with or without patch graft; subclavian, innominate, by thoracic incision
	35321	Thromboendarterectomy, with or without patch graft; axillary-brachial
	35331	Thromboendarterectomy, with or without patch graft; abdominal aorta
	35341	Thromboendarterectomy, with or without patch graft; mesenteric, celiac, or renal
	35351	Thromboendarterectomy, with or without patch graft; iliac
	35450	Transluminal balloon angioplasty, open; renal or other visceral artery
	35452	Transluminal balloon angioplasty, open; aortic
	35458	Transluminal balloon angioplasty, open; brachiocephalic trunk or branches, each vessel
	35460	Transluminal balloon angioplasty, open; venous
	35471	Transluminal balloon angioplasty, percutaneous; renal or visceral artery
	35472	Transluminal balloon angioplasty, percutaneous; aortic
	35475	Transluminal balloon angioplasty, percutaneous; brachiocephalic trunk or branches, each vessel
	35476	Transluminal balloon angioplasty, percutaneous; venous
	35480	Transluminal peripheral atherectomy, open; renal or other visceral artery
	35481	Transluminal peripheral atherectomy, open; aortic
	35484	Transluminal peripheral atherectomy, open; brachiocephalic trunk or branches, each vessel
	35490	Transluminal peripheral atherectomy, percutaneous; renal or other visceral artery
	35491	Transluminal peripheral atherectomy, percutaneous; aortic
	35494	Transluminal peripheral atherectomy, percutaneous; brachiocephalic trunk or branches, each vessel
	35500	Harvest of upper extremity vein, one segment, for lower extremity or coronary artery bypass procedure (list separately in addition to code forprimary procedure)

	Procedure Code	Code Description
	35572	Harvest of femoropopliteal vein, one segment, for vascular reconstruction procedure (eg, aortic, vena caval, coronary, peripheral artery) (listseparately in addition to code for primary procedure)
	35681	Bypass graft; composite, prosthetic and vein (list separately in addition to code for primary procedure)
	35682	Bypass graft; autogenous composite, two segments of veins from two locations (list separately in addition to code for primary procedure)
	35683	Bypass graft; autogenous composite, three or more segments of vein from two or more locations (list separately in addition to code for primaryprocedure)
	36013	Introduction of catheter, right heart or main pulmonary artery
	36470	Injection of sclerosing solution; single vein
	36471	Injection of sclerosing solution; multiple veins, same leg
	36822	Insertion of cannula(s) for prolonged extracorporeal circulation for cardiopulmonary insufficiency (ecmo) (separate procedure)
	75756	Angiography, internal mammary, radiological supervision and interpretation
	92950	Cardiopulmonary resuscitation (eg, in cardiac arrest)
	92953	Temporary transcutaneous pacing
	92960	Cardioversion, elective, electrical conversion of arrhythmia; external
	92961	Cardioversion, elective, electrical conversion of arrhythmia; internal (separate procedure)
	92970	Cardioassist-method of circulatory assist; internal
	92971	Cardioassist-method of circulatory assist; external
	92973	Percutaneous transluminal coronary thrombectomy (list separately in addition to code for primary procedure)
	92978	Intravascular ultrasound (coronary vessel/graft) during diag evaluation &/ therapeutic intervention incl imaging supervision, interpret & report;initial vessel (list sep in add to code for prim proc)
	92979	Intravascular ultrasound (coronary vessel or graft) during therapeutic intervention including imaging supervision, interpretation and report; eachadditional vessel
	92980	Transcatheter placement of an intracoronary stent(s), percutaneous, with or without other therapeutic intervention, any method; single vessel
	92981	Transcatheter placement of an intracoronary stent(s), percutaneous, with or without other therapeutic intervention, any method; each additionalvessel (list separately in add to code for primary)
	92982	Percutaneous transluminal coronary balloon angioplasty; single vessel
	92984	Percutaneous transluminal coronary balloon angioplasty; each additional vessel (list separately in addition to code for primary procedure)
	92986	Percutaneous balloon valvuloplasty; aortic valve
	92986	Percutaneous balloon valvuloplasty; aortic valve
	92987	Percutaneous balloon valvuloplasty; mitral valve
	92987	Percutaneous balloon valvuloplasty; mitral valve
	92990	Percutaneous balloon valvuloplasty; pulmonary valve
	92990	Percutaneous balloon valvuloplasty; pulmonary valve

	Procedure Code	Code Description
	92992	Atrial septectomy or septostomy; transvenous method, balloon (eg, rashkind type) (includes cardiac catheterization)
	92993	Atrial septectomy or septostomy; blade method (park septostomy) (includes cardiac catheterization)
	92995	Percutaneous transluminal coronary atherectomy, by mechanical or other method, with or without balloon angioplasty; single vessel
	92996	Percutaneous transluminal coronary atherectomy, by mechanical or other method, with or without balloon angioplasty; each additional vessel (list separately in addition to code for primary procedure)
	93501	Right heart catheterization
	93505	Endomyocardial biopsy
	93508	Catheter placement in coronary artery(s), arterial coronary conduit(s), and/or venous coronary bypass graft(s) for coronary angiography withoutconcomitant left heart catheterization
	93510	Left heart catheterization, retrograde, from the brachial artery, axillary artery or femoral artery; percutaneous
	93511	Left heart catheterization, retrograde, from the brachial artery, axillary artery or femoral artery; by cutdown
	93514	Left heart catheterization by left ventricular puncture
	93524	Combined transseptal and retrograde left heart catheterization
	93526	Combined right heart catheterization and retrograde left heart catheterization
	93527	Combined right heart catheterization and transseptal left heart catheterization through intact septum (with or without retrograde left heart catheterization)
	93528	Combined right heart catheterization with left ventricular puncture (with or without retrograde left heart catheterization)
	93529	Combined right heart catheterization and left heart catheterization through existing septal opening (with or without retrograde left heart catheterization)
	93530	Right heart catheterization, for congenital cardiac anomalies
	93531	Combined right heart catheterization and retrograde left heart catheterization, for congenital cardiac anomalies
	93532	Combined right heart catheterization and transseptal left heart catheterization through intact septum with or without retrograde left heart catheterization, for congenital cardiac anomalies
	93533	Combined right heart catheterization and transseptal left heart catheterization through existing septal opening, with or without retrograde left heart catheterization, for congenital cardiac anomalie
	93539	Injection procedure during cardiac catheterization; for selective opacification of arterial conduits (eg, internal mammary), whether native orusedfor bypass
	93540	Injection procedure during cardiac catheterization; for selective opacification of aortocoronary venous bypass grafts, one or more coronary arteries
	93541	Injection procedure during cardiac catheterization; for pulmonary angiography
	93542	Injection procedure during cardiac catheterization; for selective right ventricular or right atrial angiography

	Procedure Code	Code Description
	93543	Injection procedure during cardiac catheterization; for selective left ventricular or left atrial angiography
	93544	Injection procedure during cardiac catheterization; for aortography
	93545	Injection procedure during cardiac catheterization; for selective coronary angiography (injection of radiopaque material may be by hand)
	93555	Imaging supervision, interpretation and report for injection procedure(s) during cardiac catheterization; ventricular and/or atrial angiography
	93556	Imaging supervision, interpretation and report for injection procedure(s) during cardiac cath; pulmonary angiography, aortography, and/or selectivecoronary angiography inc venous bypass grafts
	93561	Indicator dilution studies such as dye or thermal dilution, including arterial and/or venous catheterization; with cardiac output measurement (separate procedure)
	93562	Indicator dilution studies such as dye or thermal dilution, including arterial and/or venous catheterization; subsequent measurement of cardiacoutput
	93571	Intravascular doppler velocity and/or pressure derived coronary flow reserve measurement (coronary vessel or graft) during coronary angiographyincl pharmacologically induced stress; initial vessel
	93572	Intravascular doppler velocity and/or pressure derived coronary flow reserve measurement (coronary vessel or graft) during coronary angiographyincluding pharmacologically induced stress; ea add vess
	93580	Percutaneous transcatheter closure of congenital interatrial communication (ie, fontan fenestration, atrial septal defect) with implant
	93581	Percutaneous transcatheter closure of a congenital ventricular septal defect with implant
	93600	Bundle of his recording
	93602	Intra-atrial recording
	93603	Right ventricular recording
	93609	Intraventricular &/ intra-atrial mapping, tachycardia site(s) with catheter manipulation to record,multiple sites to identify origin, tachycardia(list separately addition to code, primary procedure)
	93610	Intra-atrial pacing
	93612	Intraventricular pacing
	93613	Intracardiac electrophysiologic 3-dimensional mapping (list separately in addition to code for primary procedure)
	93618	Induction of arrhythmia by electrical pacing
	93619	Compr electrophysiologic eval w right atrial pacing&rec, right ventricular pacing&rec, his bundle rec, incl insertion&repstn, multi electrode catheters, wo induction/attempted induction, arrhythmia
	93620	Compr electrophysiologic eval incl insertion&reposition, multi electrode catheters w induction/attempted induction, arrhythmia; w right atrialpacing&rec, right ventricular pacing&rec, his bundle
	93621	Comprehensive electrophysiologic eval includ insertion & repos of mult electrode cath w induction / attempt induction of arrhythmia; w left atrialpacing & recording from coronary sinus /left atri

	Procedure Code	Code Description
	93622	Comprehensive electrophysiologic eval including insertion & repositioning of multiple electrode catheters w induction / attempted induction ofarrhythmia;w left ventricular pacing & recording
	93623	Programmed stimulation and pacing after intravenous drug infusion (list separately in addition to code for primary procedure)
	93624	Electrophysiologic follow-up study with pacing and recording to test effectiveness of therapy, including induction or attempted induction of arrhythmia
	93631	Intra-operative epicardial and endocardial pacing and mapping to localize the site of tachycardia or zone of slow conduction for surgical correction
	93640	Electrophysiologic eval,1/2 chamb pac cardiovert-defibrill leads incl defibrill thresh eval (induct of arrhythmia, eval of sensing & pacing forarrhythmia termination) at time of init implant/replace
	93641	Electrophysiologic eval,1/2 chamb pac cardiovert-defibrill leads incl defibrill thresh eval (induct of arrhythmia,sens & pac,term) time of initimplnt/replace;test of 1/2 ch pac cardiov-def pulse gen
	93642	Electrophysiologic eval, 1/2 chamb pac cardiovert-defibrill (incl defibrill thresh eval, induct of arrhythmia, eval of sens & pacing for arrhythmia term, & program/reprog of sens/therap parameters)
	93650	Intracardiac catheter ablation of atrioventricular node function, atrioventricular conduction for creation of complete heart block, with or without temporary pacemaker placement
	93650	Intracardiac catheter ablation of atrioventricular node function, atrioventricular conduction for creation of complete heart block, with or without temporary pacemaker placement
	93651	Intracardiac catheter ablation of arrhythmogenic focus; for tx of supraventricular tachycardia by ablation of fast/slow atrioventricular pathways,accessory connections or other atrial foci
	93651	Intracardiac catheter ablation of arrhythmogenic focus; for tx of supraventricular tachycardia by ablation of fast/slow atrioventricular pathways,accessory connections or other atrial foci
	93652	Intracardiac catheter ablation of arrhythmogenic focus; for treatment of ventricular tachycardia
	93652	Intracardiac catheter ablation of arrhythmogenic focus; for treatment of ventricular tachycardia
	93662	Intracardiac echocardiography during therapeutic/diagnostic intervention, including imaging supervision and interpretation (list separately inaddition to code for primary procedure)
	93701	Bioimpedance, thoracic, electrical
	93727	Electronic analysis of implantable loop recorder (ilr) system (includes retrieval of recorded and stored ecg data, physician review and interpretation of retrieved ecg data and reprogramming)
	93731	Electronic analysis of dual-chamber pacemaker, recording and interpretation at rest and exercise, analysis of event markers & device response);without reprogramming

	Procedure Code	Code Description
	93732	Electronic analysis of dual-chamber pacemaker, recording & interpret at rest and during exercise analysis of event markers and device response); with reprogramming
	93733	Electronic analysis of dual-chamber internal pacemake (may include rate, pulse amplitude and duration, configuration of wave form, and/or testingofsensory function of pacemaker) telephonic analysis
	93734	Electronic analysis of single chamber pacemaker system (inc eval of programmable parameters, record & interpret at rest & exercise, analysis ofevent markers & device response); without reprogramming
	93735	Electronic analysis of single chamber pacemaker system (inc eval of programmable parameters, record & interpret at rest & exercise, analysis ofevent markers & device response); with reprogramming
	93736	Electronic analysis of single chamber internal pacemaker (rate, pulse amplitude & duration, configuration of wave form, and/or test of sensoryfunction of pacemaker), telephonic analysis
	93741	Electronic analysis of pacing cardioverter-defibrillator (interrog,eval pulse generator,prog parameters at rest & dur act,electrocard rec & interpre of rec at rest & dur exer); 1 chamb, wo reprogram
	93742	Electronic analysis of pacing cardioverter-defibrillator (interrog,eval pulse generator,prog parameters at rest & dur act,electrocard rec & interpre of rec at rest & dur exer); 1 chamb,with reprogram
	93743	Electronic analysis of pacing cardioverter-defibrillator (interrog,eval pulse generator,prog parameters at rest & dur act,electrocard rec & interpre of rec at rest & dur exer); 2 chamber,wo reprogram
	93744	Electronic analysis of pacing cardioverter-defibrillator (interrog,eval pulse generator,prog parameters at rest & dur act,electrocard rec & interpre of rec at rest & dur exer); 2 chamber,w reprogram
	0001T	Endovascular repair of infrarenal abdominal aortic aneurysm or dissection; modular bifurcated prosthesis (two docking limbs)
	0002T	Endovascular repair of infrarenal abdominal aortic aneurysm or dissection; aorto-uni-iliac or aorto-unifemoral prosthesis
	0024T	Non-surgical septal reduction therapy (eg, alcohol ablation), for hypertrophic obstructive cardiomyopathy; with coronary arteriograms,with or without temporary pacemaker
	0033T	Endovascular repair of descending thoracic aortic aneurysm, pseudoaneurysm or dissection; involving coverage of left subclavian artery origin,initial endoprosthesis
	0034T	Endovascular repair of descending thoracic aortic aneurysm, pseudoaneurysm or dissection; not involving coverage of left subclavian artery origin,initial endoprosthesis
	0035T	Placement of proximal or distal extension prosthesis for endovascular repair of descending thoracic aortic aneurysm, pseudoaneurysm or dissection;initial extension
	0036T	Placement of proximal/ distal extension prosthesis for endovascular repair of descending thoracic aortic aneurysm, pseudoaneurysm /dissection;each additional extension

	Procedure Code	Code Description
	0318T	Transcatheter aortic valve replacement apical
	G0166	External counterpulsation, per treatment session
	G0290	Transcatheter placement of a drug eluting intracoronary stent(s), percutaneous, w/ wo other therapeutic intervention, any method; single v
	G0291	Transcatheter placement of a drug eluting intracoronary stent(s), percutaneous,w/wo other therapeutic intervention, any method; each addit
Vascular Procedures (VP)		
	ICD-9	
	00.40	Procedure-one vessel (begin 2005)
	00.41	Procedure-two vessels (begin 2005)
	00.42	Procedure-three vessels (begin 2005)
	00.43	Procedure-four+ vessels (begin 2005)
	00.44	Proc-vessel bifurcation (begin 2006)
	00.45	Insert 1 vascular stent (begin 2005)
	00.46	Insert 2 vascular stents (begin 2005)
	00.47	Insert 3 vascular stents (begin 2005)
	00.48	Insert 4+ vasculr stents (begin 2005)
	00.55	Insert drug-eluting noncrnry artry stnt (begin 2002)
	00.60	Ins d-e stnt sup fem art (begin 2010)
	17.56	Ather oth non-cor vessel (begin 2011)
	17.71	Non-coronary ifva (begin 2010)
	38.00	Incision of vessel nos
	38.01	Intracran vessel incis
	38.02	Head/neck ves incis nec
	38.03	Upper limb vessel incis
	38.05	Thoracic vessel inc nec
	38.06	Abdomen artery incision
	38.07	Abdominal vein incision
	38.08	Embolectomy leg vessel
	38.09	Lower limb vein incision
	38.10	Endarterectomy nos
	38.11	Endarter intracranial
	38.12	Head & neck endarter nec
	38.13	Upper limb endarterectom
	38.18	Endarterectomy leg vesl
	38.21	Blood vessel biopsy
	38.22	Percutaneous angioscopy (begin 1986)
	38.23	Intravasclr spectroscopy (begin 2008)
	38.24	Intravas img cor ves oct (begin 2009)
	38.25	Intravas img non-cor oct (begin 2009)

	Procedure Code	Code Description
	38.29	Blood vessel dx proc nec
	38.30	Vessel resect/anast nos
	38.31	Intracran ves resec-anas
	38.32	Head/neck ves resec-anas
	38.33	Arm vessel resect/anast
	38.35	Thor vessel resect/anast
	38.36	Abd vessel resect/anast
	38.37	Abd vein resect & anast
	38.38	Leg artery resect/anast
	38.39	Leg vein resect/anastom
	38.40	Vessel resect/replac nos
	38.41	Intracran ves resec-repl
	38.42	Head/neck ves resec-repl
	38.43	Arm ves resect w replace
	38.45	Resect thorac ves w repl (begin 1986)
	38.46	Abd artery resec w repla
	38.47	Abd vein resect w replac
	38.48	Leg artery resec w repla
	38.49	Leg vein resect w replac
	38.50	Varicose v lig-strip nos
	38.51	Intcran var v lig-strip
	38.52	Head/neck var v lig-str
	38.53	Arm varicose v lig-strip
	38.55	Thorac var v lig-strip
	38.57	Abd varicos v liga-strip
	38.59	Leg varicos v liga-strip
	38.60	Excision of vessel nos
	38.61	Intracran vessel excis
	38.62	Head/neck vessel excis
	38.63	Arm vessel excision
	38.65	Thoracic vessel excision
	38.66	Abdominal artery excis
	38.67	Abdominal vein excision
	38.68	Leg artery excision
	38.69	Leg vein excision
	38.7	Interruption vena cava
	38.80	Surg vessel occlus nec
	38.81	Occlus intracran ves nec
	38.82	Occlus head/neck ves nec
	38.83	Occlude arm vessel nec

	Procedure Code	Code Description
	38.85	Occlude thoracic ves nec
	38.86	Occlude abd artery nec
	38.87	Occlude abd vein nec
	38.88	Occlude leg artery nec
	38.89	Occlude leg vein nec
	38.91	Arterial catheterization
	38.92	Umbilical vein cath
	38.93	Other venous cath (nec) (begin 1989)
	38.97	Cv cath plcmt w guidance (begin 2010)
	39.0	Systemic-pulm art shunt
	39.1	Intra-abd venous shunt
	39.22	Aorta-subclv-carot bypas
	39.24	Aorta-renal bypass
	39.25	Aorta-iliac-femor bypass
	39.26	Intra-abdomin shunt nec
	39.28	Extracran-intracr bypass (begin 1991)
	39.29	Vasc shunt & bypass nec
	39.30	Suture of vessel nos
	39.31	Suture of artery
	39.32	Suture of vein
	39.41	Postop vasc op hem contr
	39.49	Vasc proc revision nec
	39.50	Angioplast/atherect (begin 1995)
	39.51	Clipping of aneurysm
	39.52	Aneurysm repair nec
	39.53	Arterioven fistula rep
	39.54	Re-entry operation
	39.55	Reimplan aberr renal ves
	39.56	Repair vess w tis patch
	39.57	Rep vess w synth patch
	39.58	Repair vess w patch nos
	39.59	Repair of vessel nec
	39.7	Periarter sympathectomy
	39.72	Endovas repair/occl head & neck vessels (begin 2002)
	39.74	Endo rem obs hd/neck ves (begin 2006)
	39.75	Endo emb hd/nk
	39.76	Endo em hd/nk
	39.77	Temp endovsc occls vessl (begin 2011)
	39.79	Other endovascular repair of aneurysm (begin 2000)
	39.8	Vascular body operations (end 2010)

	Procedure Code	Code Description
	39.81	Imp crtd sinus stmtotl (begin 2010)
	39.90	Insert noncor art stent (begin 1996)
	39.91	Freeing of vessel
	39.92	Vein inject-scleros agnt
	39.93	Insert ves-to-ves cannul
	39.94	Replac ves-to-ves cannul
	39.98	Hemorrhage control nos
	39.99	Vessel operation nec
	88.40	Contrast arteriogram nos
	88.42	Contrast aortogram
	88.43	Contr pulmon arteriogram
	88.44	Contr thor arteriogr nec
	88.45	Contrast renal arteriogr
	88.47	Contr abd arteriogrm nec
	88.49	Contrast arteriogram nec
	88.51	Vena cav angiocardiogram
	88.60	Contrast phlebogram nos
	88.61	Contr phlebogram-hd/neck
	88.62	Contr phlebogram-pulmon
	88.63	Thorac contr phlebog nec
	88.64	Portal contr phlebogram
	88.65	Abd contr phlebogram nec
	88.66	Contrast phlebogram-leg
	88.67	Contrast phlebogram nec
	88.68	Impedance phlebogram
	CPT	
	35400	Angioscopy (non-coronary vessels or grafts) during therapeutic intervention (list separately in addition to code for primary procedure)
	35875	Thrombectomy of arterial or venous graft (other than hemodialysis graft or fistula)
	35876	Thrombectomy of arterial or venous graft; with revision of arterial or venous graft
	35879	Revision, lower extremity arterial bypass, without thrombectomy, open; with vein patch angioplasty
	35881	Revision, lower extremity arterial bypass, without thrombectomy, open; with segmental vein interposition
	36002	Injection procedures (eg, thrombin) for percutaneous treatment of extremity pseudoaneurysm
	36005	Injection procedure for extremity venography
	36468	Single or multiple injections of sclerosing solutions, spider veins (telangiectasia); limb or trunk
	36469	Single or multiple injections of sclerosing solutions, spider veins (telangiectasia); face

	Procedure Code	Code Description
	37205	Transcatheter placement of an intravascular stent(s), (non-coronary vessel), percutaneous; initial vessel
	37206	Transcatheter placement of an intravascular stent(s), (non-coronary vessel), percutaneous; each additional vessel (list separately in additiontocode for primary procedure)
	37207	Transcatheter placement of an intravascular stent(s), (non-coronary vessel), open; initial vessel
	37208	Transcatheter placement of an intravascular stent(s), (non-coronary vessel), open; each additional vessel (list separately in addition to codeforprimary procedure)
	37620	Interruption, partial or complete, of inferior vena cava by suture, ligation, plication, clip, extravascular, intravascular (umbrella device)
	37650	Ligation of femoral vein
	37660	Ligation of common iliac vein
	37799	Unlisted procedure, vascular surgery
	50100	Transection or repositioning of aberrant renal vessels (separate procedure)
	75605	Aortography, thoracic, by serialography, radiological supervision and interpretation
	75625	Aortography, abdominal, by serialography, radiological supervision and interpretation
	75630	Aortography, abdominal plus bilateral iliofemoral lower extremity, catheter, by serialography, radiological supervision and interpretation
	75635	Computd tomograph angiography,abdomin aorta & bilatrl iliofemoral low extremity runoff,radlgicl suprvsn & intr pretatn, wo contrast material,fllowcontrst mtrial & furthr sect,incl imge post-process
	75741	Angiography, pulmonary, unilateral, selective, radiological supervision and interpretation
	75743	Angiography, pulmonary, bilateral, selective, radiological supervision and interpretation
	75746	Angiography, pulmonary, by nonselective catheter or venous injection, radiological supervision and interpretation
	75825	Venography, caval, inferior, with serialography, radiological supervision and interpretation
	75827	Venography, caval, superior, with serialography, radiological supervision and interpretation
	78445	Non-cardiac vascular flow imaging (ie, angiography, venography)
	G0269	Placement of occlusive device into either a venous or arterial access site, post surgical or interventional procedure (e.g. angioseal plug
	G0288	Reconstruction, computed tomographic angiography of aorta for surgical planning for vascular surgery
	M0301	Fabric wrapping of abdominal aneurysm (mnp)
	S2130	Endoluminal radiofrequency ablation of refluxing saphenous vein
